# The importance of baseline health in linking life purpose to longevity

**DOI:** 10.1371/journal.pone.0349401

**Published:** 2026-05-21

**Authors:** Richard Sias, Harry Turtle

**Affiliations:** 1 Department of Finance, Eller College of Management, University of Arizona, Tucson, Arizona, United States of America; 2 Department of Finance and Real Estate, College of Business, Colorado State University, Fort Collins, Colorado, United States of America; Independent Medical Researcher and Writer, UNITED KINGDOM OF GREAT BRITAIN AND NORTHERN IRELAND

## Abstract

The well-established inverse relation between purpose in life and mortality risk is widely interpreted in the literature as evidence that higher purpose causes greater longevity. This study assesses the role of baseline health in linking purpose and longevity. Based on a prospective cohort sample of 5,953 US adults aged ≥ 50 drawn from the Health and Retirement Study (2006–2018), we replicate a recent high-quality study that documents the correlation between purpose and longevity and then examine the sensitivity of the results to varying the amount of baseline health measurement error and limiting the sample to individuals with more homogeneous levels of baseline health. The relation between life purpose and longevity strongly attenuates when using more robust measures of baseline health and excluding individuals who are already seriously ill. For instance, in a model that uses the coarse measures of baseline health typically used in this literature, individuals with low purpose exhibit a hazard ratio for the subsequent 4-year period of 2.59 (95% CI = 1.43, 4.68). Once adjusting for more robust objective measures of baseline health, the hazard ratio falls to 1.77 (95% CI = 0.97, 3.22). After adjusting for objective measures of baseline health and excluding already ill individuals, the associated hazard ratio falls to 1.20 (95% CI = 0.66, 2.20) for survival over years 5–8 and 0.75 (95% CI = 0.42, 1.32) for survival over years 9–12. The results suggest that baseline health confounding or mediation explains much of the apparent relation between life purpose and longevity, and that the strong associations reported in the literature may largely reflect inadequate measurement of baseline health rather than a causal effect of purpose on longevity.

## Introduction

Purpose in life—the perception that one’s life has meaning, direction, and goals—is a psychological dimension of wellbeing typically measured via the Ryff and Keyes 7-question instrument [1]. Longevity, measured here as all-cause mortality over a 12-year follow-up period, represents the most fundamental health outcome of interest. Physical health—including chronic conditions, functional limitations, and objective measures such as lung function and grip strength—is both a determinant of longevity and a potential source of confounding in studies of psychological predictors of mortality. Purpose, longevity, and health are deeply intertwined: physical health directly affects longevity, physical health shapes psychological states including one’s sense of purpose, and purpose may influence biological processes or health behaviors that affect longevity. Understanding the nature of these interrelationships has significant implications for public health—if life purpose causally improves longevity, it represents a potentially important and low-cost lever for intervention.

Motivated by the potential public health implications of a causal purpose-longevity relation, previous work documents a remarkably strong association between life purpose and longevity [2–19]. A 2016 meta-analysis, for instance, reports that higher purpose is associated with a relative risk of 0.83 for all-cause mortality (p < 0.001) [20]. The purpose-longevity association might arise, however, because (i) high purpose causes lower future mortality risk, or (ii) a decline in baseline health (that, by definition, causes higher mortality risk) either confounds the purpose-longevity relation by causing individuals to respond more negatively to questions about purpose (e.g., “I enjoy making plans for the future and working to make them a reality”) or mediates it by serving as the pathway through which purpose affects longevity. Inconsistent with the confounding or mediation explanations, the literature concludes that purpose has a strong independent effect on longevity beyond baseline health. That is, baseline health confounding or mediation appears to play little role in explaining the purpose-mortality association, as extant work finds purpose predicts longevity after controlling for “baseline health,” “baseline physical health,” and “medical status…and health behaviors” [2,4,8]. For example, a recent study estimates that a two standard deviation shift in purpose is associated with more than 8 years of additional life and that this relation is “… substantially unchanged …” when adjusting for “… health behaviors, activity limitations, and health conditions …” [18].

A concern with the interpretation that purpose causes longevity beyond baseline health, however, is that this conclusion only holds if baseline health is adequately measured. If baseline health is coarsely measured, the correlation between life purpose and longevity may arise because purpose proxies for additional variation in baseline health. That is, if baseline health either confounds or mediates the purpose-mortality relation, then mismeasurement in baseline health will inflate the purpose-longevity relation [21]. This study’s aim is to investigate the role of baseline health mismeasurement in driving the purpose-longevity association by adopting two standard approaches for dealing with a confounding variable: reducing baseline health measurement error and restricting the sample to individuals who begin with more homogeneous baseline health levels [21,22]. If, as the literature suggests, purpose predicts longevity when controlling for baseline health, then the hazard ratios associated with purpose should largely be invariant to variation in measurement error in baseline health or the exclusion of already ill individuals.

## Materials and methods

### Study design

This study uses a prospective explanatory cohort design to examine the relation between 2006 life purpose, 2006 baseline health, and all-cause mortality over the subsequent 12 years (2006–2018). This design follows a defined population forward in time to assess the effect of an exposure on an outcome while accounting for potential confounding variables [23]. A STROBE checklist is provided in the supporting information [S1 in S1 File].

### Data source

The Health and Retirement Study (HRS) is a nationally representative biennial prospective cohort survey of individuals aged ≥ 50 and their spouses, conducted by the University of Michigan with funding from the National Institute on Aging. HRS methods and design are described elsewhere [24,25] and are available at https://hrs.isr.umich.edu/documentation/survey-design. In 2006, HRS randomly selected half the participants for an enhanced face-to-face interview that included objective measures of physical health—including lung function and grip strength—and a leave-behind psychosocial questionnaire that assessed life purpose. We use the psychosocial sample weights that adjust for both core sample weights and psychosocial sample non-response [26].

The sample consists of 8,425 individuals who were eligible to complete the questionnaire of which 7,282 had life purpose data. Inclusion criteria required participants to be aged ≥ 50, eligible for the 2006 leave-behind psychosocial questionnaire, and have non-zero psychosocial sample weights. Exclusion criteria included missing covariates, missing sample weights, or unavailable follow-up data. A total of 1,329 individuals were excluded due to missing covariates or weights, leaving an initial sample of 5,953 individuals for the 2006–2010 period. Another 132 and 302 respondents were lost due to missing follow-up data for the 2010–2014 and 2014–2018 periods, respectively. Detailed descriptions of the data cleaning process are provided in the supporting information [S2 Fig 1, S3 Table 1, S4 Table 2, and S5 Table 3 in S1 File]. The sample size for this study was determined by the HRS sampling design and the availability of complete data rather than a prospective power calculation, as is standard for secondary analyses of existing cohort data. Because this study uses deidentified publicly available information and HRS respondents provide informed written consent, it was exempted from University of Arizona’s Institutional Review Board (IRB) review.

### Data collection

HRS data collection is conducted by trained interviewers who receive standardized training for both the core interviews and the enhanced face-to-face interviews. Data collection includes demographic data, health behaviors, self-reported health conditions, objective physical health measures (including lung function assessed via Mini-Wright Peak Flow Meter to measure peak expiratory flow in liters per minute and grip strength based on a “Smedley spring-type” hand dynamometer), and psychosocial assessments including purpose in life. Full descriptions of all baseline health variables are provided in the supporting information [S6 in S1 File]. Mortality data are collected via next-of-kin exit interviews and a search of the National Death Index [27].

### Exposures and outcomes

The primary exposure is life purpose measured at baseline (2006) assessed by the Ryff and Keyes 7-question measure (e.g., “I am an active person in carrying out the plans I set for myself”) on a six-point Likert scale [1]. Following HRS guidance, we reverse score the 4 negative statements and average scores over the statements for respondents who answer ≥ 4 statements [26]. Following Alimujiang et al. [2], respondents are partitioned into five groups by life purpose score (1.00–2.99, 3.00–3.99, 4.00–4.99, 5.00–5.99, and 6.0). Additional covariates include gender, age, education, race, marital status, and four health behaviors—smoking status, alcohol consumption, vigorous physical activities, and BMI. Full variable construction details including raw HRS data names and sources are provided in supporting information S7 Table 4 in S1 File. Descriptive statistics are provided in supporting information S8 Table 5 in S1 File.

The primary outcome is all-cause mortality over the 12-year follow-up period partitioned into three successive 4-year subperiods: years 1–4 (2006–2010), years 5–8 (2010–2014), and years 9–12 (2014–2018).

The key potential confounding variable is baseline physical health in 2006. The models we consider vary with respect to baseline health metrics. The first two baseline health measures are identical to those employed by Alimujiang et al. [2]—an indicator for the presence of one or more of six chronic diseases (hypertension, diabetes, cancer, lung disease, heart disease, or stroke based on self-reported physician diagnosis) and a functional status score that ranges from 0 to 5 representing the number of activities of daily living with which the individual has some difficulty (walking across a room, bathing, eating, dressing, and getting in and out of bed). We consider five additional baseline health metrics. First, we replace the chronic disease indicator with indicators for each of the six chronic diseases as the hazard ratios vary dramatically across the conditions (S9 Table 6 in S1 File). Second, we construct a “broad limitations” metric as the first principal component from the four RAND-calculated functional limitations metrics based on whether respondents have some difficulty with Mobility, Large Muscles, Gross Motor Skills, and Fine Motor Skills (higher value implies greater physical limitations; see S10 Table 7 in S1 File) [28]. The first principal component, with eigenvalue 2.63, explains 66% of the total variation across the four functional limitation measures; all four indicators load positively on this component, with factor loadings ranging from 0.65 to 0.91. Third and fourth, we include age- and gender-adjusted lung function and grip strength. Fifth, we include respondents’ subjective self-rated health in 2006. We rescale functional limitations, broad limitations, lung function, grip strength, and self-rated health to z-scores for ease of comparison.

### Definition of terms

*Life purpose* refers to the perception that one’s life has meaning, direction, and goals and is measured by the Ryff and Keyes 7-question measure (e.g., “I am an active person in carrying out the plans I set for myself”) on a six-point Likert scale where higher values indicate greater purpose [1].

*Longevity* is operationalized as survival time in months from the 2006 baseline interview, with all-cause mortality as the event of interest. Follow-up extends over a 12-year period from 2006 to 2018.

*Baseline health* refers to physical health status at the 2006 interview and includes both self-reported indicators (e.g., diagnosed with a chronic condition and functional limitations) and objective measures (e.g., lung function and grip strength). Baseline health is a potential confounder of the purpose-longevity association if poor health causes both higher mortality risk and more negative responses to purpose questions, and a potential mediator if purpose affects longevity through its effects on baseline physical health. Our prospective cohort study framework does not differentiate between these two explanations as both produce observationally equivalent patterns of attenuation; we address this issue in detail in the Discussion section.

### Statistical analysis

We consider two standard approaches to evaluating baseline health as a potential confounder or mediator of the purpose-longevity relation. First, we estimate a series of four Cox proportional hazard models that vary the precision in baseline health measurement. Model 1 is identical to the model in Alimujiang et al. [2] except it excludes their two measures of baseline health—functional status and the chronic condition indicator. Model 2 is identical to the authors’ primary model, adding functional status and the chronic condition indicator to our Model 1. Model 3 makes two changes to Model 2: replacing the chronic condition indicator with indicators for each of the six chronic conditions and adding broad limitations, lung function, and grip strength as covariates. Model 4 adds the subjective measure of baseline health (self-rated health) to Model 3. For completeness, we also report results for Model 2, 3, and 4 without life purpose covariates to focus solely on the relation between health covariates and mortality [see S11-S13, Tables 8–10 in S1 File].

Second, following previous work that demonstrates an early mortality exclusion is an effective method of reducing the bias when the confounding variable is baseline health, we exclude individuals who perish soon after baseline to remove both diagnosed and potentially subclinical undiagnosed individuals who are seriously ill at baseline, resulting in a more homogeneous baseline health sample [21,29]. We evaluate the proportional hazards assumption using two complementary approaches. First, for each of the four model specifications, we add an interaction term between continuous life purpose score and the natural logarithm of elapsed time, estimated over the full 2006–2018 follow-up period (see S14 Table 11 in S1 File). The interaction term is statistically significant (at the *p* < 0.001 level), confirming the proportional hazards assumption is violated as the association between purpose and longevity materially weakens over time regardless of the level of health adjustment. Second, we examine Schoenfeld residual plots for the baseline Model 2 specification for each of the three 4-year subperiods (see S15 Fig 2 in S1 File). The LOESS smoother shows a noticeable upward drift over the initial 4-year window (2006–2010), a continued upward trend at the beginning of the second 4-year window (2010–2014), and a relatively flat line in the final 4-year window (2014–2018). The pattern is consistent with baseline health confounding being strongest in the near term when individuals who are already ill (both diagnosed and undiagnosed) are most likely to die at elevated rates, and suggests the proportional hazards violation attenuates as the early mortality exclusion progressively removes the most seriously ill individuals from the data and the sample becomes more homogeneous. Both tests therefore support the split-period design and are consistent with baseline health confounding being most severe in the near term. Thus, we estimate separate Cox models for three 4-year periods: years 1–4 (2006–2010), 5–8 (2010–2014), and 9–12 (2014–2018). All analyses were conducted using SAS version 9.4 (SAS Institute, Cary, NC).

## Results

Table 1 reports the results. Baseline health mismeasurement is reduced moving from Model 1 to Model 4. Results for the initial 4-year period (2006–2010, years 1–4), the subsequent 4-year period (2010–2014, years 5–8) which, by definition, is limited to individuals who survive the initial 4-year period, and final 4-year period (2014–2018, years 9–12) which is limited to individuals who survive at least 8 years post baseline, are reported, respectively, in the top, middle, and bottom portions of Table 1.

**Table 1:** HRs and 95% CIs of all-cause mortality.

	Model 1	Model 2	Model 3	Model 4
	HR (95% CI)	HR (95% CI)	HR (95% CI)	HR (95% CI)
Period: 2006–2010 (years 1–4, *n* = 5,953 individuals and 600 deaths)				
1.00-2.99 (lowest)	**3.38 (1.88-6.07)**	**2.59 (1.43-4.68)**	1.77 (0.97-3.22)	1.54 (0.85-2.82)
3.00-3.99	**2.01 (1.17-3.44)**	**1.83 (1.06-3.13)**	1.36 (0.79-2.34)	1.24 (0.72-2.14)
4.00-4.99	1.66 (0.97-2.83)	1.62 (0.95-2.76)	1.29 (0.75-2.21)	1.19 (0.70-2.04)
5.00-5.99	1.08 (0.62-1.88)	1.08 (0.62-1.88)	0.98 (0.56-1.70)	0.91 (0.52-1.59)
6.00 (reference)	1	1	1	1
Chronic disorders	None	Chronic Indicator	Individual disorders	Individual disorders
Functional score		**1.23 (1.16-1.30)**	0.99 (0.90-1.09)	1.00 (0.91-1.11)
Broad limitations			**1.24 (1.09-1.41)**	1.13 (0.99-1.30)
Lung function			**0.76 (0.69-0.84)**	**0.78 (0.71-0.86)**
Grip strength			**0.82 (0.75-0.91)**	**0.83 (0.76-0.91)**
Self-rated health				**0.76 (0.68-0.85)**
Period: 2010–2014 (years 5–8, *n* = 5,221 individuals and 702 deaths)				
1.00-2.99 (lowest)	1.78 (0.99-3.21)	1.58 (0.87-2.86)	1.20 (0.66-2.20)	1.08 (0.59-1.98)
3.00-3.99	**1.97 (1.22-3.20)**	**1.88 (1.16-3.05)**	1.58 (0.97-2.57)	1.46 (0.89-2.37)
4.00-4.99	1.33 (0.82-2.16)	1.33 (0.82-2.15)	1.19 (0.73-1.93)	1.11 (0.69-1.81)
5.00-5.99	1.52 (0.94-2.47)	1.55 (0.95-2.51)	1.48 (0.91-2.41)	1.40 (0.86-2.28)
6.00 (reference)	1	1	1	1
Chronic disorders	None	Chronic Indicator	Individual disorders	Individual disorders
Functional score		**1.18 (1.11-1.25)**	0.98 (0.89-1.09)	1.00 (0.90-1.10)
Broad limitations			**1.23 (1.09-1.39)**	1.14 (1.00-1.29)
Lung function			**0.77 (0.70-0.84)**	**0.77 (0.71-0.85)**
Grip strength			**0.85 (0.78-0.93)**	**0.86 (0.78-0.93)**
Self-rated health				**0.81 (0.73-0.90)**
Period: 2014–2018 (years 9–12, *n* = 4,217 individuals and 756 deaths)				
1.00-2.99 (lowest)	0.86 (0.49-1.50)	0.84 (0.48-1.48)	0.75 (0.42-1.32)	0.68 (0.38-1.20)
3.00-3.99	1.18 (0.80-1.73)	1.15 (0.78-1.69)	1.04 (0.71-1.54)	0.97 (0.66-1.43)
4.00-4.99	0.96 (0.66-1.40)	0.96 (0.66-1.40)	0.93 (0.64-1.36)	0.88 (0.60-1.28)
5.00-5.99	0.88 (0.60-1.29)	0.88 (0.60-1.30)	0.92 (0.63-1.35)	0.89 (0.60-1.31)
6.00 (reference)	1	1	1	1
Chronic disorders	None	Chronic Indicator	Individual disorders	Individual disorders
Functional score		1.08 (1.00-1.16)	0.97 (0.86-1.10)	0.99 (0.87-1.12)
Broad limitations			1.12 (0.97-1.28)	1.01 (0.87-1.17)
Lung function			**0.81 (0.74-0.88)**	**0.82 (0.75-0.90)**
Grip strength			**0.87 (0.80-0.95)**	**0.87 (0.80-0.96)**
Self-rated health				**0.78 (0.70-0.87)**

Note: Boldface indicates statistical significance (*p* < 0.05). Functional score, broad limitations, lung function, grip strength, and self-rated health are standardized. Additional covariates (in all models) include controls for age, sex, education, marital status, smoking status, frequency of vigorous physical activity, days/week consuming alcohol, and body mass index. All variables are measured at baseline (2006). HR, hazard ratio.

The patterns in Table 1—both across models and over horizons—are striking. First, for individuals in the lowest purpose category, there is a monotonic decline in hazard ratios associated with purpose when baseline health mismeasurement is mitigated (i.e., moving from Model 1 to Model 4) holding period constant, and over horizon holding model constant (moving from years 1−4 to years 5−8 to years 9−12). Second, the magnitude of the reduction is substantial. For instance, moving from Model 1 to Model 3, the magnitude of the effect size associated with the lowest purpose category falls 53% when examining survival in the first 4 years (i.e., ln(1.77)/ln(3.38)-1) and 68% for years 5−8 (i.e., ln(1.20)/ln(1.78)-1). Third, holding the model constant, the early mortality exclusion also results in a precipitous fall in the magnitude of the hazard ratio associated with low life purpose. For example, in Model 1, the magnitude of the low life purpose hazard ratio falls 53% (ln(1.78)/ln(3.38)-1) when simply limiting the sample to those who survive at least 4 years post baseline and falls more than 100% (i.e., the hazard ratio drops to < 1) when limiting the sample to those who survive at least 8 years post baseline.

Fourth, consistent with mismeasurement of baseline health, functional score predicts longevity only when excluding the more robust objective baseline health metrics. That is, functional score is statistically meaningful only in Model 2 for years 1–4 (HR = 1.23; 95% CI = 1.16, 1.30) and 5–8 (HR = 1.18; 95% CI = 1.11, 1.25). Once incorporating broad limitations, lung function, and grip strength (i.e., Models 3 and 4), functional score is no longer meaningfully related to survival in years 1–4 (HR = 0.99; 95% CI 0.90, 1.09) or years 5–8 (HR = 0.98; 95% CI = 0.89, 1.09). In contrast, broad limitations, lung function, and grip strength continue to meaningfully predict mortality risk. For instance, even after conditioning on survival for at least 4 years post baseline, higher broad limitations (HR = 1.23; 95% CI = 1.09, 1.39), lower lung function (HR = 0.77; 95% CI = 0.70, 0.84), and lower grip strength (HR = 0.85; 95% CI = 0.78, 0.93) all predict greater mortality risk in years 5–8.

To better gauge the potential clinical relevance, we estimate absolute risk estimates for the typical woman and man in our data (see S16 Text 2 and S17 Fig 3 in S1 File). Consistent with the pattern in hazard ratios reported in Table 1, the difference in absolute risks is greatly attenuated when either including the additional health metrics or introducing an early mortality exclusion. For instance, absent any health measures (Model 1), the difference in absolute risks for the typical woman in our sample over the first four years (years 1–4) in the lowest and highest life purpose categories is 4.9% (7.1% for low purpose versus 2.2% for high purpose). The gap between the purpose groups, however, systematically declines as additional health metrics are added to the analysis. Moving from Model 1 to Model 4, for example, the gap falls from 4.9% to 1.1%. Further consistent with the results in Table 1, the patterns in absolute risk greatly attenuate once conditioning on surviving at least 4 or 8 years post baseline. For instance, once conditioning the sample to those that survive at least 4 years post-baseline, the point estimates suggest the typical woman in the lowest life purpose category has lower absolute risk than the typical woman in the second highest life purpose category (based on Models 3 or 4).

We also examine the sensitivity of the baseline health estimates to the inclusion of purpose. Specifically, S11 Table 8, S12 Table 9, and S13 Table 10 in S1 File report hazard ratios for the baseline health metrics for Models 2, 3, and 4, respectively, both with and without the life purpose covariates. Two findings are noteworthy. First, baseline health strongly and independently predicts longevity across all model specifications and time periods. For example, when excluding purpose from Model 3, lung function (HR = 0.76; 95% CI = 0.69, 0.83), grip strength (HR = 0.81; 95% CI = 0.74, 0.90), and broad limitations (HR = 1.27; 95% CI = 1.11, 1.44) all strongly predict mortality risk in years 1–4. Second, the baseline health metric hazard ratios are essentially unchanged whether or not life purpose is included in the model. For instance, the health metric coefficients in Model 3 shift by less than 0.01 in almost all cases across all three periods. The results confirm that although adding baseline health greatly attenuates the purpose-longevity association, adding purpose has essentially no effect on the health-longevity relation.

Our tests in Table 1 follow the approach in Alimujiang et al. [2], which uses life purpose categories rather than a continuous variable to avoid imposing a specific functional form on the relation between life purpose and mortality risk. In the supporting information [see S18, S19 in S1 File], we report results after replacing the purpose categorical variable with a continuous purpose variable. These tests lead to the same conclusions—the relation between purpose and longevity is greatly attenuated when better controlling for baseline health and when employing an early mortality exclusion.

## Discussion

The purpose-mortality association systematically attenuates as measurement error in baseline health is reduced by (i) employing more robust measures of baseline health or (ii) excluding individuals who are already seriously ill. Importantly, the strategies we employ reduce, but do not eliminate, residual confounding. For example, because many seriously ill individuals survive for more than 4 or 8 years, the early mortality exclusion will not eliminate residual confounding. Similarly, although moving from Model 1 to Model 4 reduces residual confounding, the more robust baseline health metrics still contain measurement error. For instance, even with our most extended model, the framework does not (a) differentiate testicular cancer (with a 5-year survival rate in excess of 90%) from pancreatic cancer (with a 5-year survival rate less than 10%), (b) differentiate stage I lung cancer (with 5-year survival rate greater than 80%) from stage IV lung cancer (with 5-year survival rate less than 25%), or (c) classify chronic kidney disease as a chronic condition (the 5-year survival rate for a person on dialysis is 35–40%). For instance, purpose will still capture differences in health between an individual given a diagnosis with a 10% 5-year survival rate and an individual given a diagnosis with a 90% 5-year survival rate (assuming such diagnoses result in different answers to questions such as “I enjoy making plans for the future and working to make them a reality”) unless the difference in diagnoses are fully captured by broad limitations, lung function, grip strength, and self-rated health.

In short, if health impacts how respondents answer questions about purpose, then unless one can perfectly measure baseline health, residual confounding remains a potential issue. Thus, for example, an inclination to view the fact that, although the relation between the lowest purpose category and longevity is no longer statistically significant in Models 3 (HR = 1.77; 95% CI = 0.97, 3.22) and 4 (HR = 1.54; 95% CI = 0.85, 2.82) when there is no early mortality exclusion (i.e., the 2006–2010 period), the point estimates have the predicted pattern and the confidence intervals are wide, must be tempered with the knowledge that all 4 models still contain residual baseline health confounding.

Beyond the issue of residual confounding, our prospective cohort study framework does not differentiate baseline health confounding from baseline health mediation of the purpose-longevity relation, as both produce observationally identical patterns of attenuation [30]. An alternative interpretation is that baseline health mediates rather than confounds the relation between purpose and longevity. There are, however, three important considerations associated with the mediation interpretation. First, if baseline health mediates the relation, then any control for baseline health is an overadjustment and resulting purpose hazard ratios underestimate the total effect size of purpose on longevity [31,32]. Thus, under the mediation interpretation, Model 1 purpose estimates are less biased than those in Models 2, 3, and 4, and Model 1 estimates are still too conservative if the remaining covariates (e.g., BMI) also capture baseline health (S20 Table 13 in S1 File reports the hazard ratios associated with purpose excluding all control variables). Even if Model 1 estimates are too conservative, the magnitude of the life purpose causal effect is astounding under the mediation interpretation. For example, based on the Model 1 estimates (and as shown in S17 Fig 3 in S1 File), holding other factors constant, a man in the highest life purpose category exhibits an absolute mortality risk of 4.1% in years 1–4, while a man in the lowest life purpose category exhibits an absolute mortality risk triple (12.3%) that value.

Second, the mediation interpretation requires a reinterpretation of extant evidence—if baseline health fully mediates the purpose-longevity relation then, by definition, purpose does not predict longevity once controlling for baseline health as claimed in the literature. Moreover, if baseline health fully mediates the relation, then baseline health appears to be the causal channel. Thus, evidence to support other (e.g., future behavioral, biological, or stress-buffering) causal channels would require either (i) evidence that these variables mediate the purpose-longevity relation when perfectly (or at least better) controlling for baseline health (i.e., these factors are additional mediators), or (ii) evidence that these factors mediate the purpose-baseline health-longevity relation (e.g., causation runs from purpose to baseline health to future behavior to longevity).

Third, an arguably more reasonable interpretation is that baseline health both confounds and mediates the purpose-longevity relation. That is, the relation between purpose and baseline health is bidirectional [8]—purpose causes better baseline health which leads to longer lives (i.e., baseline health mediates the purpose-longevity relation) and better baseline health causes an individual to respond more favorably to questions about purpose (i.e., baseline health confounds the purpose-longevity relation). Because this is just a combination of the two explanations discussed above, this interpretation is also inconsistent with the literature’s conclusion that purpose predicts longevity after accounting for baseline health.

Perhaps more puzzling is that if the relation is bidirectional, it is so in a peculiar fashion as the evidence in Table 1 suggests that, regardless of the extent of controlling for baseline health, the purpose categories are monotonically related to longevity only in the 4 years immediately following baseline. In contrast, the Model 3 lung function hazard ratio is 0.76 (95% CI = 0.69, 0.84) for years 1–4, 0.77 (95% CI = 0.70, 0.84) for years 5–8, and 0.81 (95% CI = 0.74, 0.88) for years 9–12. Thus, under the mediation explanation, however purpose affects baseline health, that portion of baseline health caused by purpose only predicts near-term longevity. For instance, if baseline health mediates the relation between purpose and longevity because higher purpose is associated with the behavior of more frequent checkups, then the benefits of these more frequent checkups only appear in the short-term. Such an interpretation appears to contrast with evidence that the benefits of checkups primarily accrue over longer horizons [33].

Related, the early mortality exclusion may result in attenuation because purpose (either independently or through baseline health) has the strongest protective effects for individuals who do not survive at least 4 or 8 years post baseline. Nonetheless, the early mortality exclusion is a well-recognized method of controlling for baseline health in both the life purpose and broader literatures. Specifically, although several purpose-longevity studies [2,6,16] include a 1- or 2-year early mortality exclusion in their sensitivity analysis, extant work suggests that a substantially longer exclusion period is needed to better control for baseline health as most serious illnesses have survival rates beyond 1–2 years [34,35]. For example, a recent meta-analysis [36] of the relation between BMI and mortality limited the sample to 189 studies that had a minimum of 5-year early mortality exclusion to minimize baseline health confounding and a recent study [21] investigating the relation between physical activity and longevity considers a 4-year early mortality exclusion.

We also consider a wide range of sensitivity tests that demonstrate the robustness of our conclusions including a detailed investigation of the role of multicollinearity (S21, S22, S23, S24, S25, and S26 in S1 File), updating only health or purpose (S27 and S28 in S1 File), updating both health and purpose (S27 and S29 in S1 File), estimating Model 2 for the sample that does not require the additional health variables (S30 in S1 File), adding the psychological status variables used in the Alimujiang [2] extended model (S31 in S1 File), comparing the relations over years 1–2 versus 3–4 (S32 and S33 in S1 File), partitioning the sample into those with and those without a chronic condition at baseline (S34, S35, and S36 in S1 File), partitioning the sample by respondent age (S34, S37, and S38 in S1 File), and using raw (rather than age- and gender-adjusted) lung function and grip strength (S39 in S1 File). As detailed in the supporting information, regardless of how we frame the test, the results uniformly support the hypothesis that the purpose-longevity relation is greatly attenuated when minimizing measurement error in baseline health.

Our results demonstrate that the relation between purpose and longevity greatly attenuates when better capturing heterogeneity in baseline health, both through more robust baseline health measures and through an early mortality exclusion. At the same time, because our study is based on the data, methodology, and structure of a single previous study [2], it shares that study’s limitations. It is possible that alternative research designs or data may find evidence that purpose predicts longevity even when better controlling for baseline health. That is, the “absence of evidence is not evidence of absence” [37]. Nonetheless, given only coarse metrics of baseline health have been used in this literature, to claim a causal relation beyond that captured by baseline health, future work would need to demonstrate that the relation persists when better controlling for baseline health [20].

## Conclusions

Our evidence suggests the well-documented association between purpose and longevity arises from either (i) residual baseline health confounding, (ii) baseline health fully (or nearly fully) mediating the purpose-longevity relation, or (iii) some combination of (i) and (ii). All three interpretations are inconsistent with the widely held view that purpose causes longevity when controlling for baseline health.

The literature’s consensus that life purpose causes longevity appears to be so strong that recent reviews not only propose that future work should focus on understanding how purpose causes longevity (i.e., through behavioral, biological, and stress-buffering channels) rather than if purpose causes longevity, but also advise practicing health care professionals to focus on purpose to mitigate their patients’ mortality risk [5,7,8,38–43]. Moreover, governments and non-profit organizations are investing in life purpose intervention programs based on the perceived causal relation between purpose levels and subsequent morbidity. For instance, based on the interpretation in the extant literature, the American Heart Association identifies purpose (and well-being) as a new “Noteworthy” focus of their 2030 Impact Goals. Thus, understanding the causal relation between purpose, baseline health, and longevity is critical as, at best, this guidance is premature. At worst, if these findings replicate across other datasets and research designs, resources are being poorly allocated, and life purpose interventions provide potentially false guidance to patients, health care professionals, and those who influence public policy.

## Supporting information

S1 File S1 STROBE checklist. **S2 Fig 1.** Data cleaning flowchart. **S3 Table 1.** Censored and death 2006–2010. **S4 Table 2.** Censored and death 2010–2014. **S5 Table 3.** Censored and death 2014–2018. **S6 Text 1.** Baseline health variable construction. **S7 Table 4.** Variable definitions and sources. **S8 Table 5.** Descriptive characteristics of 2006 HRS participants. **S9 Table 6.** Hazard ratios for individual chronic diseases from Model 3. **S10 Table 7.** Factor loadings for broad limitations measure. **S11 Table 8.** Model 2 sensitivity of baseline health to inclusion of purpose. **S12 Table 9.** Model 3 sensitivity of baseline health to inclusion of purpose. **S13 Table 10.** Model 4 sensitivity of baseline health to inclusion of purpose. **S14 Table 11.** Constant proportionality tests. **S15 Fig 2.** Schoenfeld residual plots for life purpose score. **S16 Text 2.** Absolute risks. **S17 Fig 3.** Absolute risks for life purpose. **S18 Text 3.** Continuous life purpose. **S19 Table 12.** Continuous life purpose and mortality. **S20 Table 13.** Purpose and mortality (no covariates). **S21 Text 4.** The role of multicollinearity. **S22 Table 14.** Models 6–9 (adding health metrics one at a time). **S23 Table 15.** Standard errors for purpose (Models 0–9). **S24 Table 16.** Variance inflation factors (Models 0–9). **S25 Table 17.** Variance inflation factors for individual purpose categories. **S26 Table 18.** Variance inflation factors for purpose. **S27 Text 5.** Updating purpose and/or health. **S28 Table 19.** Model 3 updated purpose or updated baseline health. **S29 Table 20.** Models 1 and 3 with updated purpose and baseline health. **S30 Table 21.** Model 2 (includes participants without additional health metrics). **S31 Table 22.** Model 5—Adding psychological status variables to Model 4. **S32 Text 6.** Mortality in years 1–2 and 3–4. **S33 Table 23.** Life purpose and mortality (years 1–2 versus 3–4). **S34 Text 7.** Analysis by chronic condition and age. **S35 Table 24.** Models 1 and 3 for those with and without chronic condition. **S36 Table 25.** Models 1 and 3 (continuous purpose) for those with and without chronic condition. **S37 Table 26.** Models 1 and 3 for young (<75) and old (≥75). **S38 Table 27.** Models 1 and 3 (continuous purpose) for young (<75) and old (≥75). **S39 Table 28.** Models 3 and 4 based on raw lung function and grip strength. **S40** References.
(ZIP)
