# Supplementary material for: The importance of baseline health in linking life purpose to longevity
Source: PLoS One. 2026 May 21;21(5):e0349401. doi: 10.1371/journal.pone.0349401 (PMC13193554; doi:10.1371/journal.pone.0349401)
Supplement: S1 File — S2 Fig 1. Data cleaning flowchart. S3 Table 1. Censored and death 2006–2010. S4 Table 2. Censored and death 2010–2014. S5 Table 3. Censored and death 2014–2018. S6 Text 1. Baseline health variable construction. S7 Table 4. Variable definitions and sources. S8 Table 5. Descriptive characteristics of 2006 HRS participants. S9 Table 6. Hazard ratios for individual chronic diseases from Model 3. S10 Table 7. Factor loadings for broad limitations measure. S11 Table 8. Model 2 sensitivity of baseline health to inclusion of purpose. S12 Table 9. Model 3 sensitivity of baseline health to inclusion of purpose. S13 Table 10. Model 4 sensitivity of baseline health to inclusion of purpose. S14 Table 11. Constant proportionality tests. S15 Fig 2. Schoenfeld residual plots for life purpose score. S16 Text 2. Absolute risks. S17 Fig 3. Absolute risks for life purpose. S18 Text 3. Continuous life purpose. S19 Table 12. Continuous life purpose and mortality. S20 Table 13. Purpose and mortality (no covariates). S21 Text 4. The role of multicollinearity. S22 Table 14. Models 6–9 (adding health metrics one at a time). S23 Table 15. Standard errors for purpose (Models 0–9). S24 Table 16. Variance inflation factors (Models 0–9). S25 Table 17. Variance inflation factors for individual purpose categories. S26 Table 18. Variance inflation factors for purpose. S27 Text 5. Updating purpose and/or health. S28 Table 19. Model 3 updated purpose or updated baseline health. S29 Table 20. Models 1 and 3 with updated purpose and baseline health. S30 Table 21. Model 2 (includes participants without additional health metrics). S31 Table 22. Model 5—Adding psychological status variables to Model 4. S32 Text 6. Mortality in years 1–2 and 3–4. S33 Table 23. Life purpose and mortality (years 1–2 versus 3–4). S34 Text 7. Analysis by chronic condition and age. S35 Table 24. Models 1 and 3 for those with and without chronic condition. S36 Table 25. Models 1 and 3 (continuous purpose) for those with and witho [file pone.0349401.s001.zip › S34_Text.pdf]

S34 Text 7. Analysis by chronic condition and age.

Our primary analysis takes two standard approaches to reducing the potential confounding from baseline health—reducing baseline health measurement error (i.e., adding baseline health metrics) and restricting the sample to individuals who begin with more homogeneous baseline health levels (i.e., the early mortality exclusion). An alternative approach used in the literature [3,5] is to exclude individuals who, ex-ante, are more likely to perish soon after baseline by limiting the sample to individuals with no chronic illnesses. Correspondingly, work suggests that the ability of life purpose to predict longevity is stronger for older individuals [9] who, of course, are also more likely to perish soon after baseline. Thus, to limit the sample to those whose death is more "unexpected," we repeat our tests restricting the sample to (1) those with and without a chronic condition at baseline, and (2) younger (<75 years) and older (≥75 years) individuals.

S35 Table 24 reports the primary analysis (using life purpose categories as in Alimujiang et al. [3]) partitioned by individuals with no chronic conditions at baseline versus those with a chronic condition at baseline. S36 Table 25 reports the corresponding analysis using the continuous life purpose measure. Regardless of the approach (categorical life purpose or continuous life purpose), the results reveal no evidence that purpose predicts mortality when limited to individuals with no chronic diseases when better controlling for baseline health (i.e., Model 3). Objectively, when limiting the sample to individuals with no chronic diseases, the sample (especially the number of deaths), is small which likely reduces power (although point estimates should be unbiased). Further consistent with our primary results, we document the same broad patterns in both samples (those with and those without a chronic disease): the point estimates are severely attenuated when including the additional baseline health metrics or adding an early mortality exclusion.

S37 Table 26 reports the primary analysis partitioned by age (<75 and ≥75) at baseline. The results for the initial 4-year period reveal that absent health controls (i.e. Model 1), those in the lowest life purpose category exhibit meaningfully larger hazard ratios. The results, however, are substantially stronger for individuals over age 75. Once adding the health metrics (Model 3), those in the lowest life purpose category exhibit meaningfully larger hazard ratios (relative to those in the highest category) only for older (≥75) individuals and the hazard ratio is attenuated relative to the model that excludes baseline health metrics. When limited to younger (<75) individuals, the results in the third column reveal little evidence of systematic relation between purpose and longevity once better controlling for baseline health even in the initial 4-year period. The results for years 5-8 and 9-12 reveal little evidence life purpose has any systematic relation with longevity for older (≥75) individuals regardless of whether the model includes any controls for baseline health. For younger individuals, however, the hazard ratio for individuals in the second lowest group (purpose between 3-3.99) is meaningfully different from the reference of 1.00. However, there is little evidence of a systematic relation between purpose and longevity as, for example, the second *highest* life purpose category exhibits the second greatest hazard ratio in years 5-8.

S38 Table 27 reports the corresponding analysis (young versus old) using the continuous life purpose measure and reveals similar results. Once controlling for baseline health, (1) there is little evidence that purpose predicts longevity for individuals under age 75 at any horizon, and (2) evidence of a relation between purpose and longevity for older individuals is limited to the near-term (years 1-4).
