# Supplementary material for: The importance of baseline health in linking life purpose to longevity
Source: PLoS One. 2026 May 21;21(5):e0349401. doi: 10.1371/journal.pone.0349401 (PMC13193554; doi:10.1371/journal.pone.0349401)
Supplement: S1 File — S2 Fig 1. Data cleaning flowchart. S3 Table 1. Censored and death 2006–2010. S4 Table 2. Censored and death 2010–2014. S5 Table 3. Censored and death 2014–2018. S6 Text 1. Baseline health variable construction. S7 Table 4. Variable definitions and sources. S8 Table 5. Descriptive characteristics of 2006 HRS participants. S9 Table 6. Hazard ratios for individual chronic diseases from Model 3. S10 Table 7. Factor loadings for broad limitations measure. S11 Table 8. Model 2 sensitivity of baseline health to inclusion of purpose. S12 Table 9. Model 3 sensitivity of baseline health to inclusion of purpose. S13 Table 10. Model 4 sensitivity of baseline health to inclusion of purpose. S14 Table 11. Constant proportionality tests. S15 Fig 2. Schoenfeld residual plots for life purpose score. S16 Text 2. Absolute risks. S17 Fig 3. Absolute risks for life purpose. S18 Text 3. Continuous life purpose. S19 Table 12. Continuous life purpose and mortality. S20 Table 13. Purpose and mortality (no covariates). S21 Text 4. The role of multicollinearity. S22 Table 14. Models 6–9 (adding health metrics one at a time). S23 Table 15. Standard errors for purpose (Models 0–9). S24 Table 16. Variance inflation factors (Models 0–9). S25 Table 17. Variance inflation factors for individual purpose categories. S26 Table 18. Variance inflation factors for purpose. S27 Text 5. Updating purpose and/or health. S28 Table 19. Model 3 updated purpose or updated baseline health. S29 Table 20. Models 1 and 3 with updated purpose and baseline health. S30 Table 21. Model 2 (includes participants without additional health metrics). S31 Table 22. Model 5—Adding psychological status variables to Model 4. S32 Text 6. Mortality in years 1–2 and 3–4. S33 Table 23. Life purpose and mortality (years 1–2 versus 3–4). S34 Text 7. Analysis by chronic condition and age. S35 Table 24. Models 1 and 3 for those with and without chronic condition. S36 Table 25. Models 1 and 3 (continuous purpose) for those with and witho [file pone.0349401.s001.zip › S40_References.pdf]

S40 References

1. Bellera CA, MacGrogan G, Debled M, de Lara CT, Brouste V, Mathoulin-Pélissier S. Variables with time-varying effects and the cox model: Some statistical concepts illustrated with a prognostic factor study in breast cancer. BMC Med Res Methodol. 2010 Mar 16;10:20. doi:10.1186/1471-2288-10-20 PubMed PMID: 20233435; PubMed Central PMCID: PMC2846954.

2. Schemper M. Cox analysis of survival data with non-proportional hazard functions. Journal of the Royal Statistical Society Series D (The Statistician). 1992;41(4):455–65. doi:10.2307/2349009

3. Alimujiang A, Wiensch A, Boss J, Fleischer NL, Mondul AM, McLean K, et al. Association between life purpose and mortality among US adults older than 50 years. JAMA Network Open. 2019 May 24;2(5):e194270. doi:10.1001/jamanetworkopen.2019.4270

4. Boylan JM, Tompkins JL, Krueger PM. Psychological well-being, education, and mortality. Health Psychology. 2022 Mar;41(3):225–34. Located at: 2022-30409-001. doi:10.1037/hea0001159

5. Angelantonio ED, Bhupathiraju SN, Wormser D, Gao P, Kaptoge S, Gonzalez AB de, et al. Body-mass index and all-cause mortality: individual-participant-data meta-analysis of 239 prospective studies in four continents. The Lancet. 2016 Aug 20;388(10046):776–86. doi:10.1016/S0140-6736(16)30175-1 PubMed PMID: 27423262.

6. Strain T, Wijndaele K, Sharp SJ, Dempsey PC, Wareham N, Brage S. Impact of follow-up time and analytical approaches to account for reverse causality on the association between physical activity and health outcomes in UK Biobank. International Journal of Epidemiology. 2020 Feb 1;49(1):162–72. doi:10.1093/ije/dyz212

7. Dattani S, Samborska V, Ritchie H, Roser M. Cancer. Our World in Data [Internet]. 2024 Oct 7 [cited 2025 Nov 10]. Available from: https://ourworldindata.org/cancer

8. Taylor CJ, Ordóñez-Mena JM, Roalfe AK, Lay-Flurrie S, Jones NR, Marshall T, et al. Trends in survival after a diagnosis of heart failure in the United Kingdom 2000-2017: Population based cohort study. BMJ. 2019 Feb 13;364:l223. doi:10.1136/bmj.l223 PubMed PMID: 30760447.

9. Tanno K, Sakata K, Ohsawa M, Onoda T, Itai K, Yaegashi Y, et al. Associations of Ikigai as a positive psychological factor with all-cause mortality and cause-specific mortality among middle-aged and elderly japanese people: Findings from the Japan collaborative cohort study. Journal of Psychosomatic Research. 2009 Jul 1;67(1):67–75. doi:10.1016/j.jpsychores.2008.10.018
