# Supplementary material for: The importance of baseline health in linking life purpose to longevity
Source: PLoS One. 2026 May 21;21(5):e0349401. doi: 10.1371/journal.pone.0349401 (PMC13193554; doi:10.1371/journal.pone.0349401)
Supplement: S1 File — S2 Fig 1. Data cleaning flowchart. S3 Table 1. Censored and death 2006–2010. S4 Table 2. Censored and death 2010–2014. S5 Table 3. Censored and death 2014–2018. S6 Text 1. Baseline health variable construction. S7 Table 4. Variable definitions and sources. S8 Table 5. Descriptive characteristics of 2006 HRS participants. S9 Table 6. Hazard ratios for individual chronic diseases from Model 3. S10 Table 7. Factor loadings for broad limitations measure. S11 Table 8. Model 2 sensitivity of baseline health to inclusion of purpose. S12 Table 9. Model 3 sensitivity of baseline health to inclusion of purpose. S13 Table 10. Model 4 sensitivity of baseline health to inclusion of purpose. S14 Table 11. Constant proportionality tests. S15 Fig 2. Schoenfeld residual plots for life purpose score. S16 Text 2. Absolute risks. S17 Fig 3. Absolute risks for life purpose. S18 Text 3. Continuous life purpose. S19 Table 12. Continuous life purpose and mortality. S20 Table 13. Purpose and mortality (no covariates). S21 Text 4. The role of multicollinearity. S22 Table 14. Models 6–9 (adding health metrics one at a time). S23 Table 15. Standard errors for purpose (Models 0–9). S24 Table 16. Variance inflation factors (Models 0–9). S25 Table 17. Variance inflation factors for individual purpose categories. S26 Table 18. Variance inflation factors for purpose. S27 Text 5. Updating purpose and/or health. S28 Table 19. Model 3 updated purpose or updated baseline health. S29 Table 20. Models 1 and 3 with updated purpose and baseline health. S30 Table 21. Model 2 (includes participants without additional health metrics). S31 Table 22. Model 5—Adding psychological status variables to Model 4. S32 Text 6. Mortality in years 1–2 and 3–4. S33 Table 23. Life purpose and mortality (years 1–2 versus 3–4). S34 Text 7. Analysis by chronic condition and age. S35 Table 24. Models 1 and 3 for those with and without chronic condition. S36 Table 25. Models 1 and 3 (continuous purpose) for those with and witho [file pone.0349401.s001.zip › S1_Strobe.pdf]

## S1 STROBE checklist.

| | Item No | Recommendation | Page No |
|---|---|---|---|
| **Title and abstract** | 1 | (*a*) Indicate the study's design with a commonly used term in the title or the abstract<br>(*b*) Provide in the abstract an informative and balanced summary of what was done and what was found | 1 |
| **Introduction** | | | |
| Background/rationale | 2 | Explain the scientific background and rationale for the investigation being reported | 2-3 |
| Objectives | 3 | State specific objectives, including any prespecified hypotheses | 2-3 |
| **Methods** | | | |
| Study design | 4 | Present key elements of study design early in the paper | 3 |
| Setting | 5 | Describe the setting, locations, and relevant dates, including periods of recruitment, exposure, follow-up, and data collection | 3-4 |
| Participants | 6 | (*a*) Give the eligibility criteria, and the sources and methods of selection of participants. Describe methods of follow-up<br>(*b*) For matched studies, give matching criteria and number of exposed and unexposed | 3<br>S2-<br>S5 |
| Variables | 7 | Clearly define all outcomes, exposures, predictors, potential confounders, and effect modifiers. Give diagnostic criteria, if applicable | 4-5<br>S2,<br>S6-<br>S7 |
| Data sources/ measurement | 8* | For each variable of interest, give sources of data and details of methods of assessment (measurement). Describe comparability of assessment methods if there is more than one group | S2,<br>S6-<br>S7 |
| Bias | 9 | Describe any efforts to address potential sources of bias | 10-<br>13,<br>S11-<br>S15,<br>S20,<br>S27-<br>S39 |
| Study size | 10 | Explain how the study size was arrived at | 3-4,<br>S2-<br>S5 |
| Quantitative variables | 11 | Explain how quantitative variables were handled in the analyses. If applicable, describe which groupings were chosen and why | 4-6,<br>S10 |
| Statistical methods | 12 | (*a*) Describe all statistical methods, including those used to control for confounding<br>(*b*) Describe any methods used to examine subgroups and interactions<br>(*c*) Explain how missing data were addressed<br>(*d*) If applicable, explain how loss to follow-up was addressed<br>(*e*) Describe any sensitivity analyses | 6-13,<br>S9,<br>S11-<br>S39 |
| **Results** | | | |
| Participants | 13* | (a) Report numbers of individuals at each stage of study—eg numbers potentially eligible, examined for eligibility, confirmed eligible, included in the study, completing follow-up, and analysed<br>(b) Give reasons for non-participation at each stage<br>(c) Consider use of a flow diagram | 3-4,<br>S3-<br>S5 |
| Descriptive data | 14* | (a) Give characteristics of study participants (eg demographic, clinical, social) and information on exposures and potential confounders | S8 |
| | | (b) Indicate number of participants with missing data for each variable of interest | S2 |

| | | | |
|---|---|---|---|
| | | (c) Summarise follow-up time (eg, average and total amount) | S3-S5 |
| Outcome data | 15* | Report numbers of outcome events or summary measures over time | 4-6, S8 |
| Main results | 16 | (a) Give unadjusted estimates and, if applicable, confounder-adjusted estimates and their precision (eg, 95% confidence interval). Make clear which confounders were adjusted for and why they were included | 7-9, S20-S22, S27-S39 |
| | | (b) Report category boundaries when continuous variables were categorized | 4-5 |
| | | (c) If relevant, consider translating estimates of relative risk into absolute risk for a meaningful time period | S16-S17 |
| Other analyses | 17 | Report other analyses done—eg analyses of subgroups and interactions, and sensitivity analyses | S9-S39 |

**Discussion**

| | | | |
|---|---|---|---|
| Key results | 18 | Summarise key results with reference to study objectives | 7-9 |
| Limitations | 19 | Discuss limitations of the study, taking into account sources of potential bias or imprecision. Discuss both direction and magnitude of any potential bias | 13 |
| Interpretation | 20 | Give a cautious overall interpretation of results considering objectives, limitations, multiplicity of analyses, results from similar studies, and other relevant evidence | 10-13 |
| Generalisability | 21 | Discuss the generalisability (external validity) of the study results | 13-14 |

**Other information**

| | | | |
|---|---|---|---|
| Funding | 22 | Give the source of funding and the role of the funders for the present study and, if applicable, for the original study on which the present article is based | n/a |
