# Supplementary material for: The importance of baseline health in linking life purpose to longevity
Source: PLoS One. 2026 May 21;21(5):e0349401. doi: 10.1371/journal.pone.0349401 (PMC13193554; doi:10.1371/journal.pone.0349401)
Supplement: S1 File — S2 Fig 1. Data cleaning flowchart. S3 Table 1. Censored and death 2006–2010. S4 Table 2. Censored and death 2010–2014. S5 Table 3. Censored and death 2014–2018. S6 Text 1. Baseline health variable construction. S7 Table 4. Variable definitions and sources. S8 Table 5. Descriptive characteristics of 2006 HRS participants. S9 Table 6. Hazard ratios for individual chronic diseases from Model 3. S10 Table 7. Factor loadings for broad limitations measure. S11 Table 8. Model 2 sensitivity of baseline health to inclusion of purpose. S12 Table 9. Model 3 sensitivity of baseline health to inclusion of purpose. S13 Table 10. Model 4 sensitivity of baseline health to inclusion of purpose. S14 Table 11. Constant proportionality tests. S15 Fig 2. Schoenfeld residual plots for life purpose score. S16 Text 2. Absolute risks. S17 Fig 3. Absolute risks for life purpose. S18 Text 3. Continuous life purpose. S19 Table 12. Continuous life purpose and mortality. S20 Table 13. Purpose and mortality (no covariates). S21 Text 4. The role of multicollinearity. S22 Table 14. Models 6–9 (adding health metrics one at a time). S23 Table 15. Standard errors for purpose (Models 0–9). S24 Table 16. Variance inflation factors (Models 0–9). S25 Table 17. Variance inflation factors for individual purpose categories. S26 Table 18. Variance inflation factors for purpose. S27 Text 5. Updating purpose and/or health. S28 Table 19. Model 3 updated purpose or updated baseline health. S29 Table 20. Models 1 and 3 with updated purpose and baseline health. S30 Table 21. Model 2 (includes participants without additional health metrics). S31 Table 22. Model 5—Adding psychological status variables to Model 4. S32 Text 6. Mortality in years 1–2 and 3–4. S33 Table 23. Life purpose and mortality (years 1–2 versus 3–4). S34 Text 7. Analysis by chronic condition and age. S35 Table 24. Models 1 and 3 for those with and without chronic condition. S36 Table 25. Models 1 and 3 (continuous purpose) for those with and witho [file pone.0349401.s001.zip › S5_Table.pdf]

S5 Table 3. Censored and death 2014-2018.

Dead or alive in 2018	N	Case	Death/censored date
Died 2006-2010 (1)	600		Died prior to 2010 interview
Died 2010-2014 (2)	702		Died prior to 2014 interview
Died 2014-2018 (3)	79	Reported as died during 2018 wave, no death date given	Assumed death date=July 2017
Died 2014-2018 (4)	677	Reported died with death date	Reported death date
Alive (5)	3,100	Interviewed in 2018 wave	Censored 2018 interview date
Alive (6)	361	Last interviewed in 2016 wave	Censored 2016 interview date
Unknown (7)	192	Last interviewed in 2014 wave	Lacks follow up data
Unknown (8)	110	Last interviewed in 2012 wave	Lacks follow up data
Unknown (9)	69	Last interviewed in 2010 wave	Lacks follow up data
Unknown (10)	63	Last interviewed in 2008 wave	Lacks follow up data
2014-2018 sample	4,217	(3) + (4) + (5) + (6)	
Total	5,953	(1) + (2) + (3) + (4) + (5) + (6) + (7) + (8) + (9) + (10)	
