# Supplementary material for: The importance of baseline health in linking life purpose to longevity
Source: PLoS One. 2026 May 21;21(5):e0349401. doi: 10.1371/journal.pone.0349401 (PMC13193554; doi:10.1371/journal.pone.0349401)
Supplement: S1 File — S2 Fig 1. Data cleaning flowchart. S3 Table 1. Censored and death 2006–2010. S4 Table 2. Censored and death 2010–2014. S5 Table 3. Censored and death 2014–2018. S6 Text 1. Baseline health variable construction. S7 Table 4. Variable definitions and sources. S8 Table 5. Descriptive characteristics of 2006 HRS participants. S9 Table 6. Hazard ratios for individual chronic diseases from Model 3. S10 Table 7. Factor loadings for broad limitations measure. S11 Table 8. Model 2 sensitivity of baseline health to inclusion of purpose. S12 Table 9. Model 3 sensitivity of baseline health to inclusion of purpose. S13 Table 10. Model 4 sensitivity of baseline health to inclusion of purpose. S14 Table 11. Constant proportionality tests. S15 Fig 2. Schoenfeld residual plots for life purpose score. S16 Text 2. Absolute risks. S17 Fig 3. Absolute risks for life purpose. S18 Text 3. Continuous life purpose. S19 Table 12. Continuous life purpose and mortality. S20 Table 13. Purpose and mortality (no covariates). S21 Text 4. The role of multicollinearity. S22 Table 14. Models 6–9 (adding health metrics one at a time). S23 Table 15. Standard errors for purpose (Models 0–9). S24 Table 16. Variance inflation factors (Models 0–9). S25 Table 17. Variance inflation factors for individual purpose categories. S26 Table 18. Variance inflation factors for purpose. S27 Text 5. Updating purpose and/or health. S28 Table 19. Model 3 updated purpose or updated baseline health. S29 Table 20. Models 1 and 3 with updated purpose and baseline health. S30 Table 21. Model 2 (includes participants without additional health metrics). S31 Table 22. Model 5—Adding psychological status variables to Model 4. S32 Text 6. Mortality in years 1–2 and 3–4. S33 Table 23. Life purpose and mortality (years 1–2 versus 3–4). S34 Text 7. Analysis by chronic condition and age. S35 Table 24. Models 1 and 3 for those with and without chronic condition. S36 Table 25. Models 1 and 3 (continuous purpose) for those with and witho [file pone.0349401.s001.zip › S6_Text.pdf]

S6 Text 1. Baseline health variable construction.

Background for Physical Measurements: Beginning in 2006, HRS began an assessment of physical performance for respondents selected for face-to-face interviews. Respondents in nursing homes, those who had a proxy respondent, and those who were only able to do a telephone interview were not given the physical tests. Respondents were asked to read and waive a consent form and, prior to each test, were asked if they understood the directions and felt safe. Those who did not understand or did not feel safe were not given the test. The process included nine physical measurements: blood pressure, pulse, lung function, hand grip strength, balance tests (which varied by age), timed walk test (for those age 65 and older), height, weight, and waist circumference. Given (i) blood pressure is often controlled by medications, (ii) pulse or weight loss/gain may indicate either health improvement or health deterioration, and (iii) the walk speed and balance tests were only given to a subset of respondents or the test varied by respondent age, we focus on the lung function and grip tests.

Lung Function Test: Interviewers used a Mini-Wright Peak Flow Meter with a disposable mouthpiece to measure peak expiratory flow (the amount and rate that air can be pushed out of the lungs after a full inhalation) in liters per minute. Respondents were given three attempts (with at least 30 seconds between attempts). We use the variables R8PUFF (the maximum value from the three tests) for 2006 from the RAND 1992-2018 longitudinal file. We sort respondents who have a 2006 lung test by gender and age group (50-54, 55-59, 60-64, 65-69, 70-74, 75-79, 80+) and then compute each respondent's age- and gender-adjusted lung function as their value less the average value for those in the same age/gender group. We standardize the resulting age- and gender-adjusted lung function score for ease in interpretation.

Grip Test: Using a "Smedley spring-type" hand dynamometer, respondents are asked to squeeze the dynamometer as hard as they can. Respondents are given four attempts—two attempts for their dominant hand and two attempts for their non-dominant hand. We use the variables R8GRP (the maximum value from the four grip tests) for 2006 from the RAND 1992-2018 longitudinal file. We follow a process identical to that for lung function (see above for detail) to form age- and gender-adjusted measures of grip strength.

Hypertension Diagnosis: At each core interview wave (i.e., every two years), respondents are asked about a hypertension diagnosis. If it is the respondent's first interview, they are asked, "Has a doctor ever told you that you have high blood pressure or hypertension?" If it is not the respondent's first interview and the respondent previously reported they had hypertension, the respondent is told, "Our records from your last interview [in [previous interview wave month] [previous interview wave year]] show that you have had high blood pressure or hypertension" but interviewers record if the respondent disputes this information. If a respondent was previously interviewed and did not report hypertension, the respondent is asked, "Since we last talked to you [in [previous interview wave month] [previous interview wave year]], has a doctor told you that you have high blood pressure or hypertension?" Similarly, respondents are allowed to dispute the previous record. We use the variables R8HIBPE for 2006 from the RAND 1992-2018 longitudinal file.

Diabetes Diagnosis: Analogous to hypertension diagnosis, respondents are asked, "… has a doctor told you that you have diabetes or high blood sugar?" We use the variables R8DIABE for 2006 from the RAND 1992-2018 longitudinal file.

Cancer Diagnosis: Analogous to hypertension diagnosis, respondents are asked, "… has a doctor told you that you have cancer or a malignant tumor, excluding minor skin cancer?" We use the variables R8CANCRE for 2006 from the RAND 1992-2018 longitudinal file.

Lung Disease Diagnosis: Analogous to hypertension diagnosis, respondents are asked, "… has a doctor told you that you have chronic lung disease such as chronic bronchitis or emphysema?" We use the variables R8LUNGE for 2006 from the RAND 1992-2018 longitudinal file.

Heart Condition Diagnosis: Analogous to hypertension diagnosis, respondents are asked, "… has a doctor told you that you have had a heart attack, (have) coronary heart disease, angina, congestive heart failure, or other heart problems?" We use the variables R8HEARTE for 2006 from the RAND 1992-2018 longitudinal file.

Stroke Diagnosis: Analogous to hypertension diagnosis, respondents are asked, "… has a doctor told you that you have had a stroke?" We use the variables R8STROKE for 2006 from the RAND 1992-2018 longitudinal file.

Broad limitations calculation: We compute broad limitations as the first principal component of the four RAND functional limitation metrics: Mobility (R8MOBILA), Large Muscle (R8LGMUSA), Gross Motor Skills (R8GROSSA), and Fine Motor Skills (R8FINEA). Each index is a sum of the number of functions that the respondent has "some difficulty." Mobility includes walking one block, walking several blocks, walking across a room, climbing one flight of stairs, and climbing several flights of stairs. Large muscle includes sitting for two hours, getting up from a chair, stooping, kneeling or crouching, and pushing or pulling large objects activities. The gross motor skills include walking one block, walking across a room, climbing one flight of stairs, getting in or out of bed, and bathing activities. Fine motor skills include picking up a dime, eating, and dressing. The first principal component explains 66% of the total variation in the four factors and has an eigenvalue of 2.63.

Self rated health: Respondents are asked, "Would you say your health is excellent, very good, good, fair, or poor?" We reverse score the variable so higher values indicate better health. We use the variable R8SHLT for 2006 from the RAND 1992-2018 longitudinal file.
