# Supplementary material for: The importance of baseline health in linking life purpose to longevity
Source: PLoS One. 2026 May 21;21(5):e0349401. doi: 10.1371/journal.pone.0349401 (PMC13193554; doi:10.1371/journal.pone.0349401)
Supplement: S1 File — S2 Fig 1. Data cleaning flowchart. S3 Table 1. Censored and death 2006–2010. S4 Table 2. Censored and death 2010–2014. S5 Table 3. Censored and death 2014–2018. S6 Text 1. Baseline health variable construction. S7 Table 4. Variable definitions and sources. S8 Table 5. Descriptive characteristics of 2006 HRS participants. S9 Table 6. Hazard ratios for individual chronic diseases from Model 3. S10 Table 7. Factor loadings for broad limitations measure. S11 Table 8. Model 2 sensitivity of baseline health to inclusion of purpose. S12 Table 9. Model 3 sensitivity of baseline health to inclusion of purpose. S13 Table 10. Model 4 sensitivity of baseline health to inclusion of purpose. S14 Table 11. Constant proportionality tests. S15 Fig 2. Schoenfeld residual plots for life purpose score. S16 Text 2. Absolute risks. S17 Fig 3. Absolute risks for life purpose. S18 Text 3. Continuous life purpose. S19 Table 12. Continuous life purpose and mortality. S20 Table 13. Purpose and mortality (no covariates). S21 Text 4. The role of multicollinearity. S22 Table 14. Models 6–9 (adding health metrics one at a time). S23 Table 15. Standard errors for purpose (Models 0–9). S24 Table 16. Variance inflation factors (Models 0–9). S25 Table 17. Variance inflation factors for individual purpose categories. S26 Table 18. Variance inflation factors for purpose. S27 Text 5. Updating purpose and/or health. S28 Table 19. Model 3 updated purpose or updated baseline health. S29 Table 20. Models 1 and 3 with updated purpose and baseline health. S30 Table 21. Model 2 (includes participants without additional health metrics). S31 Table 22. Model 5—Adding psychological status variables to Model 4. S32 Text 6. Mortality in years 1–2 and 3–4. S33 Table 23. Life purpose and mortality (years 1–2 versus 3–4). S34 Text 7. Analysis by chronic condition and age. S35 Table 24. Models 1 and 3 for those with and without chronic condition. S36 Table 25. Models 1 and 3 (continuous purpose) for those with and witho [file pone.0349401.s001.zip › S7_Table.pdf]

S7 Table 4. Variable definitions and sources.

Variable	Variable ID/construction	Source
Leave behind questionnaire weights	KLBWGTR	HRS Tracker file
Death date	RADDATE	RAND longitudinal file
Interview status	I8WBEG, I9WBEG, I10WBEG, I11WBEG, I12WBEG, I13WBEG, I14WBEG	RAND longitudinal file
Age (years)	R8AGEY_B	RAND longitudinal file
Sex	RAGENDER	RAND longitudinal file
Marital status	R8MSTAT<br>Married: R8MSTAT=1, 2, or 3<br>Divorced: R8MSTAT=4, 5, or 6<br>Widowed: R8MSTAT=7<br>Never married: R8MSTAT=8	RAND longitudinal file
Race/ethnicity	RACACEM and RAHISPAN<br>White: RACACEM=1 and RAHISPAN=0<br>Black: RACEM=2<br>Hispanic White: RACACEM=1 RAHISPN=1<br>Other: RARACEM=3	RAND longitudinal file
Education level	RAEDUC and RAEDEGRM<br>Less than high school: RAEDUC=1<br>High school graduate: RAEDUC=2 or RAEDUC=3<br>Some college: RAEDUC=4<br>College: RAEDUC=5 and RAEDEGRM NE 6 or 7<br>Graduate degree: RAEDEGRM=6 or 7<br>We use the RAND definitions for educational level. RAEDUC identify less than high school, GED, high school graduate, some college, and college and above. RAND defines some college as more than 12 years of education and has a high school diploma or GED or if the degree is less than a BA or "other." RAEDEGRM is highest degree earned. We denote MA/MBA and Law/MD/PhD as graduate degrees.	RAND longitudinal file
Smoking status	R8SMOKEN and R8SMOKEV<br>Current smoker: R8SMOKEN=1<br>Former smoker: R8SMOKEN NE 1 and R8SMOKEV=1<br>Never smoker: R8SMOKEV=0	RAND longitudinal file
Alcohol consumption	R8DRINKD<br>0: R8DRINKD=0<br>1-2: R8DRINKD=1 or R8DRINKD=2<br>3-4: R8DRINKD=3 or R8DRINKD=4<br>5-6: R8DRINKD=5 or R8DRINKD=6<br>7: R8DRINKD=7	RAND longitudinal file
BMI	R8BMI<br>Low BMI: R8BMI<18·5<br>Normal BMI: 18·5 ≤R8BMI<25<br>Overweight BMI: 25≤R8BMI<30<br>Obese BMI: R8BMI>30	RAND longitudinal file

Variable	Variable ID/Construction	Source
Vigorous physical exercise	R8VGACTX<br>Daily: R8VGACTX=1<br>>1/week: R8VGACTX=2<br>1/week: R8VGACTX=3<br>1-3/month: R8VGACTX=4<br>Hardly ever or never: R8VGACTX=5	RAND longitudinal file
High blood pressure	R8HIPBE=1	RAND longitudinal file
Diabetes	R8DIABE=1	RAND longitudinal file
Cancer	R8CANCRE=1	RAND longitudinal file
Lung disease	R8LUNGE=1	RAND longitudinal file
Heart disease	R8HEARTE=1	RAND longitudinal file
Stroke	R8STROKE=1	RAND longitudinal file
Chronic illness	If any of the above (R8HBIPE, R8DIABE, R8CANCRE, R8LUNGE, R8HEARTE, or R8STOKEK), equals 1, chronic disease=1	RAND longitudinal file
Functional score	R8ADLA	RAND longitudinal file
Life purpose score	For individuals who answer at least four of the seven questions:<br>LP=sum(KLB035A,-1*KLB035B,KLB035C,-1*KLB035D,<br>-1*KLB035E,-1*KLB035F,KLB035G)	RAND 2006 fat file
Self-rate health	R8SHLT<br>Self-rated health=6-R8SHLT	RAND longitudinal file
Broad limitations	First principal component of R8MOBILA, R8LGMUSA, R8GROSSA, and R8FINEA	RAND longitudinal file
Grip strength	R8GRP	RAND longitudinal file
Lung function	R8PUFF	RAND longitudinal file
