# Supplementary material for: The importance of baseline health in linking life purpose to longevity
Source: PLoS One. 2026 May 21;21(5):e0349401. doi: 10.1371/journal.pone.0349401 (PMC13193554; doi:10.1371/journal.pone.0349401)
Supplement: S1 File — S2 Fig 1. Data cleaning flowchart. S3 Table 1. Censored and death 2006–2010. S4 Table 2. Censored and death 2010–2014. S5 Table 3. Censored and death 2014–2018. S6 Text 1. Baseline health variable construction. S7 Table 4. Variable definitions and sources. S8 Table 5. Descriptive characteristics of 2006 HRS participants. S9 Table 6. Hazard ratios for individual chronic diseases from Model 3. S10 Table 7. Factor loadings for broad limitations measure. S11 Table 8. Model 2 sensitivity of baseline health to inclusion of purpose. S12 Table 9. Model 3 sensitivity of baseline health to inclusion of purpose. S13 Table 10. Model 4 sensitivity of baseline health to inclusion of purpose. S14 Table 11. Constant proportionality tests. S15 Fig 2. Schoenfeld residual plots for life purpose score. S16 Text 2. Absolute risks. S17 Fig 3. Absolute risks for life purpose. S18 Text 3. Continuous life purpose. S19 Table 12. Continuous life purpose and mortality. S20 Table 13. Purpose and mortality (no covariates). S21 Text 4. The role of multicollinearity. S22 Table 14. Models 6–9 (adding health metrics one at a time). S23 Table 15. Standard errors for purpose (Models 0–9). S24 Table 16. Variance inflation factors (Models 0–9). S25 Table 17. Variance inflation factors for individual purpose categories. S26 Table 18. Variance inflation factors for purpose. S27 Text 5. Updating purpose and/or health. S28 Table 19. Model 3 updated purpose or updated baseline health. S29 Table 20. Models 1 and 3 with updated purpose and baseline health. S30 Table 21. Model 2 (includes participants without additional health metrics). S31 Table 22. Model 5—Adding psychological status variables to Model 4. S32 Text 6. Mortality in years 1–2 and 3–4. S33 Table 23. Life purpose and mortality (years 1–2 versus 3–4). S34 Text 7. Analysis by chronic condition and age. S35 Table 24. Models 1 and 3 for those with and without chronic condition. S36 Table 25. Models 1 and 3 (continuous purpose) for those with and witho [file pone.0349401.s001.zip › S8_Table.pdf]

S8 Table 5. Descriptive characteristics of 2006 HRS participants.

Characteristic	Participants, No. (%) 2006-2010		Participants, No. (%) 2010-2014		Participants, No. (%) 2014-2018	
	No event (*n*=5,353)	Death (*n*=600)	No event (*n*=4,591)	Death (*n*=702)	No event (*n*=3,461)	Death (*n*=756)
Age, y						
50-54	420 (7.8)	9 (1.5)	390 (8.6)	17 (2.4)	347 (10)	13 (1.7)
55-59	866 (16.2)	27 (4.5)	814 (18)	32 (4.6)	716 (20.7)	38 (5)
60-64	833 (15.6)	40 (6.7)	765 (16.9)	46 (6.6)	654 (18.9)	68 (9)
65-69	1102 (20.6)	92 (15.3)	965 (21.4)	103 (14.7)	774 (22.4)	134 (17.7)
70-74	911 (17)	97 (16.2)	769 (17)	123 (17.5)	554 (16)	155 (20.5)
75-79	632 (11.8)	102 (17)	475 (10.5)	141 (20.1)	287 (8.3)	160 (21.2)
80+	589 (11)	233 (38.8)	341 (7.5)	240 (34.2)	129 (3.7)	188 (24.9)
Gender						
Male	2256 (42.1)	307 (51.2)	1838 (40.7)	362 (51.6)	1367 (39.5)	350 (46.3)
Female	3097 (57.9)	293 (48.8)	2681 (59.3)	340 (48.4)	2094 (60.5)	406 (53.7)
Marital Status						
Married	3770 (70.4)	329 (54.8)	3246 (71.8)	430 (61.3)	2546 (73.6)	478 (63.2)
Separated or divorced	575 (10.7)	53 (8.8)	494 (10.9)	64 (9.1)	385 (11.1)	72 (9.5)
Widowed	868 (16.2)	195 (32.5)	657 (14.5)	198 (28.2)	433 (12.5)	189 (25)
Never married	140 (2.6)	23 (3.8)	122 (2.7)	10 (1.4)	97 (2.8)	17 (2.2)
Race/ethnicity						
Non-Hispanic white	4252 (79.4)	490 (81.7)	3572 (79)	568 (80.9)	2705 (78.2)	620 (82)
Non-Hispanic and Hispanic black	627 (11.7)	70 (11.7)	528 (11.7)	91 (13)	410 (11.8)	97 (12.8)
Hispanic white	262 (4.9)	25 (4.2)	233 (5.2)	26 (3.7)	191 (5.5)	24 (3.2)
Other	212 (4)	15 (2.5)	186 (4.1)	17 (2.4)	155 (4.5)	15 (2)
Educational level						
<High school	853 (15.9)	169 (28.2)	677 (15)	164 (23.4)	468 (13.5)	173 (22.9)
High school	1986 (37.1)	220 (36.7)	1660 (36.7)	272 (38.7)	1250 (36.1)	289 (38.2)
Some college	1244 (23.2)	121 (20.2)	1071 (23.7)	147 (20.9)	844 (24.4)	155 (20.5)
College	750 (14)	52 (8.7)	649 (14.4)	79 (11.3)	518 (15)	87 (11.5)
Graduate school	520 (9.7)	38 (6.3)	462 (10.2)	40 (5.7)	381 (11)	52 (6.9)
Smoking Status						
Never	2404 (44.9)	195 (32.5)	2093 (46.3)	253 (36)	1627 (47)	323 (42.7)
Current smoker	654 (12.2)	95 (15.8)	531 (11.8)	103 (14.7)	396 (11.4)	105 (13.9)
Former smoker	2295 (42.9)	310 (51.7)	1895 (41.9)	346 (49.3)	1438 (41.5)	328 (43.4)
Alcohol, days drink/wk						
0	3468 (64.8)	442 (73.7)	2900 (64.2)	489 (69.7)	2186 (63.2)	525 (69.4)
1-2	896 (16.7)	85 (14.2)	782 (17.3)	93 (13.2)	632 (18.3)	98 (13)
3-4	356 (6.7)	20 (3.3)	312 (6.9)	33 (4.7)	242 (7)	42 (5.6)
5-6	186 (3.5)	9 (1.5)	167 (3.7)	15 (2.1)	134 (3.9)	21 (2.8)
Every day	447 (8.4)	44 (7.3)	358 (7.9)	72 (10.3)	267 (7.7)	70 (9.3)
Vigorous phy. exercise						
Every day	158 (3)	19 (3.2)	139 (3.1)	14 (2)	106 (3.1)	20 (2.6)
More than once a week	1212 (22.6)	66 (11)	1068 (23.6)	109 (15.5)	865 (25)	132 (17.5)
Once a week	447 (8.4)	27 (4.5)	395 (8.7)	39 (5.6)	325 (9.4)	49 (6.5)
1-3 Times a month	383 (7.2)	22 (3.7)	344 (7.6)	30 (4.3)	281 (8.1)	40 (5.3)
Hardly ever or never	3153 (58.9)	466 (77.7)	2573 (56.9)	510 (72.6)	1884 (54.4)	515 (68.1)

Characteristic	Participants, No. (%) 2006-2010		Participants, No. (%) 2010-2014		Participants, No. (%) 2014-2018	
	No event (*n*=5,353)	Death (*n*=600)	No event (n=4,591)	Death (*n*=702)	No event (*n*=3,461)	Death (*n*=756)
Chronic illness						
No	1649 (30.8)	63 (10.5)	1492 (33)	105 (15)	1241 (35.9)	142 (18.8)
Yes	3704 (69.2)	537 (89.5)	3027 (67)	597 (85)	2220 (64.1)	614 (81.2)
Body mass index						
≤ 18.50	50 (0.9)	31 (5.2)	40 (0.9)	9 (1.3)	27 (0.8)	11 (1.5)
18.51-24.99	1473 (27.5)	214 (35.7)	1200 (26.6)	228 (32.5)	881 (25.5)	234 (31)
25.00-29.99	2117 (39.5)	186 (31)	1803 (39.9)	261 (37.2)	1388 (40.1)	281 (37.2)
≥ 30.00	1713 (32)	169 (28.2)	1476 (32.7)	204 (29.1)	1165 (33.7)	230 (30.4)
Life purpose category						
1.00-2.99	164 (3.1)	54 (9)	127 (2.8)	31 (4.4)	97 (2.8)	24 (3.2)
3.00-3.99	1159 (21.7)	205 (34.2)	903 (20)	223 (31.8)	637 (18.4)	215 (28.4)
4.00-4.99	1925 (36)	222 (37)	1647 (36.4)	237 (33.8)	1262 (36.5)	273 (36.1)
5.00-5.99	1750 (32.7)	101 (16.8)	1522 (33.7)	188 (26.8)	1208 (34.9)	206 (27.2)
6.00	355 (6.6)	18 (3)	320 (7.1)	23 (3.3)	257 (7.4)	38 (5)
High blood pressure						
No	2524 (47.2)	214 (35.7)	2192 (48.5)	261 (37.2)	1753 (50.7)	288 (38.1)
Yes	2829 (52.8)	386 (64.3)	2327 (51.5)	441 (62.8)	1708 (49.3)	468 (61.9)
Diabetes						
No	4427 (82.7)	417 (69.5)	3785 (83.8)	524 (74.6)	2934 (84.8)	587 (77.6)
Yes	926 (17.3)	183 (30.5)	734 (16.2)	178 (25.4)	527 (15.2)	169 (22.4)
Cancer						
No	4630 (86.5)	440 (73.3)	3973 (87.9)	541 (77.1)	3102 (89.6)	602 (79.6)
Yes	723 (13.5)	160 (26.7)	546 (12.1)	161 (22.9)	359 (10.4)	154 (20.4)
Lung disease						
No	4981 (93.1)	480 (80)	4247 (94)	606 (86.3)	3287 (95)	674 (89.2)
Yes	372 (6.9)	120 (20)	272 (6)	96 (13.7)	174 (5)	82 (10.8)
Heart disease						
No	4268 (79.7)	323 (53.8)	3714 (82.2)	443 (63.1)	2949 (85.2)	511 (67.6)
Yes	1085 (20.3)	277 (46.2)	805 (17.8)	259 (36.9)	512 (14.8)	245 (32.4)
Stroke						
No	5047 (94.3)	503 (83.8)	4297 (95.1)	624 (88.9)	3328 (96.2)	677 (89.6)
Yes	306 (5.7)	97 (16.2)	222 (4.9)	78 (11.1)	133 (3.8)	79 (10.4)
Continuous variables: Mean (SD)						
Life purpose score	4.6 (0.9)	4.13 (0.94)	4.63 (0.89)	4.36 (0.91)	4.67 (0.88)	4.43 (0.89)
Functional score	-0.06 (0.89)	0.52 (1.59)	-0.1 (0.81)	0.23 (1.28)	-0.13 (0.78)	0.02 (0.96)
Broad limitations	-0.07 (0.93)	0.65 (1.31)	-0.14 (0.87)	0.36 (1.17)	-0.19 (0.83)	0.12 (1.01)
Adjusted hand grip	0.04 (0.99)	-0.39 (0.93)	0.08 (1)	-0.2 (0.94)	0.13 (1.01)	-0.16 (0.93)
Adjusted lung function	0.06 (0.97)	-0.57 (0.97)	0.13 (0.97)	-0.37 (0.97)	0.2 (0.95)	-0.22 (0.96)
Self-rated health	0.07 (0.97)	-0.65 (1.01)	0.14 (0.95)	-0.35 (1)	0.2 (0.93)	-0.2 (0.94)

Number of respondents in each variable response category for all variables within each HRS wave (2006-2010, 2010-2014, 2014-2018). Percent responses by variable-wave response are provided for each variable in parentheses. Because purpose and health both decline with age and mortality risk rises with age, the surviving sample's 2006 life purpose, health metrics, and mortality rates rise, and the average (2006) age declines, over the three periods.
