# Supplementary material for: The importance of baseline health in linking life purpose to longevity
Source: PLoS One. 2026 May 21;21(5):e0349401. doi: 10.1371/journal.pone.0349401 (PMC13193554; doi:10.1371/journal.pone.0349401)
Supplement: S1 File — S2 Fig 1. Data cleaning flowchart. S3 Table 1. Censored and death 2006–2010. S4 Table 2. Censored and death 2010–2014. S5 Table 3. Censored and death 2014–2018. S6 Text 1. Baseline health variable construction. S7 Table 4. Variable definitions and sources. S8 Table 5. Descriptive characteristics of 2006 HRS participants. S9 Table 6. Hazard ratios for individual chronic diseases from Model 3. S10 Table 7. Factor loadings for broad limitations measure. S11 Table 8. Model 2 sensitivity of baseline health to inclusion of purpose. S12 Table 9. Model 3 sensitivity of baseline health to inclusion of purpose. S13 Table 10. Model 4 sensitivity of baseline health to inclusion of purpose. S14 Table 11. Constant proportionality tests. S15 Fig 2. Schoenfeld residual plots for life purpose score. S16 Text 2. Absolute risks. S17 Fig 3. Absolute risks for life purpose. S18 Text 3. Continuous life purpose. S19 Table 12. Continuous life purpose and mortality. S20 Table 13. Purpose and mortality (no covariates). S21 Text 4. The role of multicollinearity. S22 Table 14. Models 6–9 (adding health metrics one at a time). S23 Table 15. Standard errors for purpose (Models 0–9). S24 Table 16. Variance inflation factors (Models 0–9). S25 Table 17. Variance inflation factors for individual purpose categories. S26 Table 18. Variance inflation factors for purpose. S27 Text 5. Updating purpose and/or health. S28 Table 19. Model 3 updated purpose or updated baseline health. S29 Table 20. Models 1 and 3 with updated purpose and baseline health. S30 Table 21. Model 2 (includes participants without additional health metrics). S31 Table 22. Model 5—Adding psychological status variables to Model 4. S32 Text 6. Mortality in years 1–2 and 3–4. S33 Table 23. Life purpose and mortality (years 1–2 versus 3–4). S34 Text 7. Analysis by chronic condition and age. S35 Table 24. Models 1 and 3 for those with and without chronic condition. S36 Table 25. Models 1 and 3 (continuous purpose) for those with and witho [file pone.0349401.s001.zip › S14_Table.pdf]

S14 Table 11. Constant proportionality tests.

	Model 1 HR (95% CI)	Model 2 HR (95% CI)	Model 3 HR (95% CI)	Model 4 HR (95% CI)
Life purpose	**0.46 (0.35-0.61)**	**0.52 (0.39-0.68)**	**0.58 (0.45-0.76)**	**0.59 (0.45-0.77)**
Life purpose × ln(1+no. months)	**1.15 (1.08-1.23)**	**1.13 (1.06-1.21)**	**1.12 (1.05-1.19)**	**1.12 (1.05-1.20)**

The Cox proportional hazard model assumes that life purpose has a constant impact on mortality risk. To examine this assumption across all four model specifications, we add a variable interacting life purpose with the natural logarithm of elapsed time, estimated over the full 2006-2018 follow-up period.[1,2] Specifically, for each model we replace the life purpose group indicator variables with the individual's life purpose score and the product of the individual's life purpose score and the natural logarithm of 1+number of months until censoring or death. A statistically significant interaction term indicates the relation between life purpose and mortality risk varies over time. Because we interact the natural logarithm of time with life purpose score (where a higher value indicates greater life purpose), a hazard ratio greater than one for the interaction term indicates the relation between purpose and mortality risk declines with time.

The interaction term is statistically significant across all four model specifications (p<0.001 in all cases), confirming the proportional hazards assumption is violated regardless of the level of baseline health adjustment. Specifically, the net log hazard ratio for life purpose at any elapsed time t (in months) is:

Estimate(life purpose) + Estimate(Life purpose × ln(1+no. months)) × ln(1+no. months)

Setting this value to zero and solving for t yields the estimated crossover point where the estimated net association between higher purpose and mortality risk would turn neutral or negative. The estimated crossover point is approximately 227 months (19 years) in Model 1, 191 months (16 years) in Model 2, 117 months (10 years) in Model 3, and 100 months (8 years) in Model 4.

Three features of these results are noteworthy. First, the crossover points for Models 1 and 2 fall well beyond our 144-month (12-year) observation window, meaning at least the point estimate of the net relation between purpose and longevity remains positive throughout our entire follow up period under these specifications.

Second, and more importantly, the compression of the crossover point (from 19 years in Model 1 to 8 years in Model 4) provides a striking quantitative illustration of our study's central point. Specifically, once baseline health is more precisely measured, not only does the relation between purpose and longevity substantially attenuate, but the crossover point moves substantially closer. The result is consistent with the hypothesis that the apparent long-term protective association in models that fail to adequately control for baseline health may be largely due to the inadequate control rather than a causal relation between purpose and longevity.

Third, abstracting from the time-varying nature of the association, the attenuation of the life purpose score hazard ratio from Model 1 (HR=0.46) to Model 4 (HR=0.59) mirrors the attenuation pattern in the main Table 1 results, confirming the central finding of the paper is robust to this alternative approach. Boldface indicates statistical significance (p<0.05).
