# Supplementary material for: The importance of baseline health in linking life purpose to longevity
Source: PLoS One. 2026 May 21;21(5):e0349401. doi: 10.1371/journal.pone.0349401 (PMC13193554; doi:10.1371/journal.pone.0349401)
Supplement: S1 File — S2 Fig 1. Data cleaning flowchart. S3 Table 1. Censored and death 2006–2010. S4 Table 2. Censored and death 2010–2014. S5 Table 3. Censored and death 2014–2018. S6 Text 1. Baseline health variable construction. S7 Table 4. Variable definitions and sources. S8 Table 5. Descriptive characteristics of 2006 HRS participants. S9 Table 6. Hazard ratios for individual chronic diseases from Model 3. S10 Table 7. Factor loadings for broad limitations measure. S11 Table 8. Model 2 sensitivity of baseline health to inclusion of purpose. S12 Table 9. Model 3 sensitivity of baseline health to inclusion of purpose. S13 Table 10. Model 4 sensitivity of baseline health to inclusion of purpose. S14 Table 11. Constant proportionality tests. S15 Fig 2. Schoenfeld residual plots for life purpose score. S16 Text 2. Absolute risks. S17 Fig 3. Absolute risks for life purpose. S18 Text 3. Continuous life purpose. S19 Table 12. Continuous life purpose and mortality. S20 Table 13. Purpose and mortality (no covariates). S21 Text 4. The role of multicollinearity. S22 Table 14. Models 6–9 (adding health metrics one at a time). S23 Table 15. Standard errors for purpose (Models 0–9). S24 Table 16. Variance inflation factors (Models 0–9). S25 Table 17. Variance inflation factors for individual purpose categories. S26 Table 18. Variance inflation factors for purpose. S27 Text 5. Updating purpose and/or health. S28 Table 19. Model 3 updated purpose or updated baseline health. S29 Table 20. Models 1 and 3 with updated purpose and baseline health. S30 Table 21. Model 2 (includes participants without additional health metrics). S31 Table 22. Model 5—Adding psychological status variables to Model 4. S32 Text 6. Mortality in years 1–2 and 3–4. S33 Table 23. Life purpose and mortality (years 1–2 versus 3–4). S34 Text 7. Analysis by chronic condition and age. S35 Table 24. Models 1 and 3 for those with and without chronic condition. S36 Table 25. Models 1 and 3 (continuous purpose) for those with and witho [file pone.0349401.s001.zip › S15_Fig.pdf]

S15 Fig 2. Schoenfeld residual plots for life purpose score.

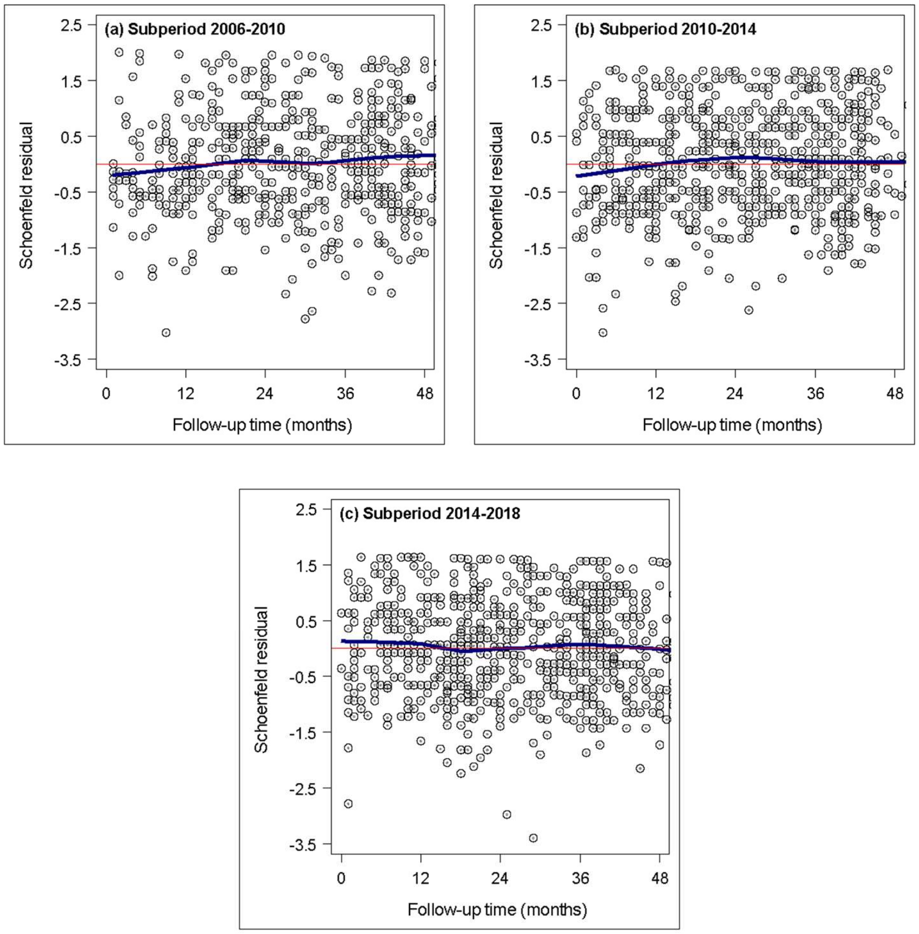

Each panel plots the Schoenfeld residual for the continuous life purpose score against follow-up time in months with a LOESS smoother (bandwidth=0.5, blue line) for Model 2. Under the proportional hazards assumption, residuals should be randomly scattered around zero with no trend over time. Panels (a)-(c) report results for each of the three 4-year subperiods.

Inconsistent with the proportional hazards assumption, panel (a) shows a noticeable upward trend in the LOESS smoother during the 2006-2010 period indicating the relation between purpose and mortality weakens over time even within the initial 4-year window. The pattern is consistent with baseline health confounding being strongest in the near term when individuals who are already ill (both diagnosed and undiagnosed) are most likely to die at elevated rates. The LOESS smoother continues to show an upward trend early in the second 4-year period (panel (b)) and is largely flat by the third 4-year period (panel (c)), suggesting the proportional hazards violation attenuates as the early mortality exclusion progressively removes the most seriously ill individuals from the data and the sample becomes more homogeneous. In sum, the results are consistent with the formal tests in S14 Table 11 that strongly reject the proportional hazards assumption and motivate the split-period design used in the main analysis.
