# Supplementary material for: The importance of baseline health in linking life purpose to longevity
Source: PLoS One. 2026 May 21;21(5):e0349401. doi: 10.1371/journal.pone.0349401 (PMC13193554; doi:10.1371/journal.pone.0349401)
Supplement: S1 File — S2 Fig 1. Data cleaning flowchart. S3 Table 1. Censored and death 2006–2010. S4 Table 2. Censored and death 2010–2014. S5 Table 3. Censored and death 2014–2018. S6 Text 1. Baseline health variable construction. S7 Table 4. Variable definitions and sources. S8 Table 5. Descriptive characteristics of 2006 HRS participants. S9 Table 6. Hazard ratios for individual chronic diseases from Model 3. S10 Table 7. Factor loadings for broad limitations measure. S11 Table 8. Model 2 sensitivity of baseline health to inclusion of purpose. S12 Table 9. Model 3 sensitivity of baseline health to inclusion of purpose. S13 Table 10. Model 4 sensitivity of baseline health to inclusion of purpose. S14 Table 11. Constant proportionality tests. S15 Fig 2. Schoenfeld residual plots for life purpose score. S16 Text 2. Absolute risks. S17 Fig 3. Absolute risks for life purpose. S18 Text 3. Continuous life purpose. S19 Table 12. Continuous life purpose and mortality. S20 Table 13. Purpose and mortality (no covariates). S21 Text 4. The role of multicollinearity. S22 Table 14. Models 6–9 (adding health metrics one at a time). S23 Table 15. Standard errors for purpose (Models 0–9). S24 Table 16. Variance inflation factors (Models 0–9). S25 Table 17. Variance inflation factors for individual purpose categories. S26 Table 18. Variance inflation factors for purpose. S27 Text 5. Updating purpose and/or health. S28 Table 19. Model 3 updated purpose or updated baseline health. S29 Table 20. Models 1 and 3 with updated purpose and baseline health. S30 Table 21. Model 2 (includes participants without additional health metrics). S31 Table 22. Model 5—Adding psychological status variables to Model 4. S32 Text 6. Mortality in years 1–2 and 3–4. S33 Table 23. Life purpose and mortality (years 1–2 versus 3–4). S34 Text 7. Analysis by chronic condition and age. S35 Table 24. Models 1 and 3 for those with and without chronic condition. S36 Table 25. Models 1 and 3 (continuous purpose) for those with and witho [file pone.0349401.s001.zip › S16_Text.pdf]

S16 Text 2. Absolute risks.

To better gauge the potential clinical relevance of purpose, we estimate absolute risk estimates for women and men (separately) with the "mode" characteristics (see S8): age group 65-69, high school graduate, non-Hispanic White race, married, never smoked, non-drinker, vigorous exercise hardly ever or never, overweight, and has high blood pressure but no other chronic disease. The graphs on the left-hand side of S17 Fig 3 report 4-year absolute mortality risk for women from each of our four models (see Table 1) for the life-purpose categories. The top, middle, and bottom figures are for years 1-4 (2006-2010), 5-8 (2010-2014), and 9-12 (2014-2018), respectively. The right-hand side presents corresponding figures for men. For ease of exposition, we simply refer to these as the "typical" woman or man.

The results are fully consistent with the hazard ratios reported in Table 1. For example, the Model 1 results in the top left graph demonstrate that, absent any health measures, the absolute risk for the typical woman in the lowest life purpose category in years 1-4 is 7.1% versus 2.2% for the typical woman in the highest life purpose category (a 4.9% difference). The gap between the purpose groups, however, systematically declines as additional health metrics are added to the analysis. For example, Model 4 of the same figure reports the absolute risk for the typical woman in the lowest life purpose category in years 1-4 is 3.1% versus 2.0% for the typical women in the highest life purpose category (a 1.1% difference). The top right-hand graph in S17 Fig 3 reports an identical pattern for the typical man.

In addition, fully consistent with our Table 1 results, the patterns (for both women and men) greatly attenuate once conditioning on surviving at least 4 (middle set of graphs) or 8 years post baseline (bottom graph set). For example, for both the typical woman and man, the point estimates in Model 3 for years 5-8 suggest that a life purpose between 5-6 is associated with a more than 1% *greater* absolute risk than individuals with a life purpose score less than 3. That is, conditional on surviving at least 4 years post baseline and once controlling for baseline health, there is no evidence the typical man or woman in the lowest life purpose group has greater absolute risk than the typical man or woman in the second highest life purpose group.
