# Supplementary material for: The importance of baseline health in linking life purpose to longevity
Source: PLoS One. 2026 May 21;21(5):e0349401. doi: 10.1371/journal.pone.0349401 (PMC13193554; doi:10.1371/journal.pone.0349401)
Supplement: S1 File — S2 Fig 1. Data cleaning flowchart. S3 Table 1. Censored and death 2006–2010. S4 Table 2. Censored and death 2010–2014. S5 Table 3. Censored and death 2014–2018. S6 Text 1. Baseline health variable construction. S7 Table 4. Variable definitions and sources. S8 Table 5. Descriptive characteristics of 2006 HRS participants. S9 Table 6. Hazard ratios for individual chronic diseases from Model 3. S10 Table 7. Factor loadings for broad limitations measure. S11 Table 8. Model 2 sensitivity of baseline health to inclusion of purpose. S12 Table 9. Model 3 sensitivity of baseline health to inclusion of purpose. S13 Table 10. Model 4 sensitivity of baseline health to inclusion of purpose. S14 Table 11. Constant proportionality tests. S15 Fig 2. Schoenfeld residual plots for life purpose score. S16 Text 2. Absolute risks. S17 Fig 3. Absolute risks for life purpose. S18 Text 3. Continuous life purpose. S19 Table 12. Continuous life purpose and mortality. S20 Table 13. Purpose and mortality (no covariates). S21 Text 4. The role of multicollinearity. S22 Table 14. Models 6–9 (adding health metrics one at a time). S23 Table 15. Standard errors for purpose (Models 0–9). S24 Table 16. Variance inflation factors (Models 0–9). S25 Table 17. Variance inflation factors for individual purpose categories. S26 Table 18. Variance inflation factors for purpose. S27 Text 5. Updating purpose and/or health. S28 Table 19. Model 3 updated purpose or updated baseline health. S29 Table 20. Models 1 and 3 with updated purpose and baseline health. S30 Table 21. Model 2 (includes participants without additional health metrics). S31 Table 22. Model 5—Adding psychological status variables to Model 4. S32 Text 6. Mortality in years 1–2 and 3–4. S33 Table 23. Life purpose and mortality (years 1–2 versus 3–4). S34 Text 7. Analysis by chronic condition and age. S35 Table 24. Models 1 and 3 for those with and without chronic condition. S36 Table 25. Models 1 and 3 (continuous purpose) for those with and witho [file pone.0349401.s001.zip › S18_Text.pdf]

## S18 Text 3. Continuous life purpose.

We follow Alimujiang et al. [3] and partition respondents into five groups by their life purpose score. In this section, we repeat our primary tests but replace the life purpose categorical variables with the continuous life purpose variables. S19 Table 12 reports the hazard ratios for the life purpose and health variables. Fully consistent with the life purpose categorical analysis, the effect size associated with life purpose is greatly attenuated once incorporating the additional baseline health measures or an early mortality exclusion. For example, nearly identical to the corresponding discussion in the paper, when moving from Model 1 to Model 3, the magnitude of the effect size associated with life purpose falls 53% when examining survival in the first 4 years (i.e., ln(0.86)/ln(0.72)-1) and 87% for years 5-8 (i.e., ln(0.98)/ln(0.88)-1). The hazard ratios for the health variables are effectively identical to their corresponding values in Table 1.

We do find, however, that in the initial 4-year period life purpose remains statistically significant at traditional levels when measured as a continuous variable (versus the life purpose categories). The results are consistent with two potential interpretations: there remains a statistically meaningful relation (albeit greatly attenuated) between purpose and mortality when fully controlling for baseline health (albeit the protective power is limited to the near term), or life purpose captures variation in baseline health not accounted for by the baseline health measures. That is, although the additional health metrics should more fully capture baseline health (relative to the measures used in most previous work), they remain crude measures. For instance, as discussed in the study, even with our most extended model, the framework does not (a) differentiate testicular cancer (with a 5-year survival rate in excess of 90%) from pancreatic cancer (with a 5-year survival rate less than 10%), (b) differentiate stage I lung cancer (with 5-year survival rate greater than 80%) from stage IV lung cancer (with 5-year survival rate less than 25%), or (c) consider chronic kidney disease as a chronic condition (the 5-year survival rate for a person on dialysis is 35-40%).
