# Supplementary material for: The importance of baseline health in linking life purpose to longevity
Source: PLoS One. 2026 May 21;21(5):e0349401. doi: 10.1371/journal.pone.0349401 (PMC13193554; doi:10.1371/journal.pone.0349401)
Supplement: S1 File — S2 Fig 1. Data cleaning flowchart. S3 Table 1. Censored and death 2006–2010. S4 Table 2. Censored and death 2010–2014. S5 Table 3. Censored and death 2014–2018. S6 Text 1. Baseline health variable construction. S7 Table 4. Variable definitions and sources. S8 Table 5. Descriptive characteristics of 2006 HRS participants. S9 Table 6. Hazard ratios for individual chronic diseases from Model 3. S10 Table 7. Factor loadings for broad limitations measure. S11 Table 8. Model 2 sensitivity of baseline health to inclusion of purpose. S12 Table 9. Model 3 sensitivity of baseline health to inclusion of purpose. S13 Table 10. Model 4 sensitivity of baseline health to inclusion of purpose. S14 Table 11. Constant proportionality tests. S15 Fig 2. Schoenfeld residual plots for life purpose score. S16 Text 2. Absolute risks. S17 Fig 3. Absolute risks for life purpose. S18 Text 3. Continuous life purpose. S19 Table 12. Continuous life purpose and mortality. S20 Table 13. Purpose and mortality (no covariates). S21 Text 4. The role of multicollinearity. S22 Table 14. Models 6–9 (adding health metrics one at a time). S23 Table 15. Standard errors for purpose (Models 0–9). S24 Table 16. Variance inflation factors (Models 0–9). S25 Table 17. Variance inflation factors for individual purpose categories. S26 Table 18. Variance inflation factors for purpose. S27 Text 5. Updating purpose and/or health. S28 Table 19. Model 3 updated purpose or updated baseline health. S29 Table 20. Models 1 and 3 with updated purpose and baseline health. S30 Table 21. Model 2 (includes participants without additional health metrics). S31 Table 22. Model 5—Adding psychological status variables to Model 4. S32 Text 6. Mortality in years 1–2 and 3–4. S33 Table 23. Life purpose and mortality (years 1–2 versus 3–4). S34 Text 7. Analysis by chronic condition and age. S35 Table 24. Models 1 and 3 for those with and without chronic condition. S36 Table 25. Models 1 and 3 (continuous purpose) for those with and witho [file pone.0349401.s001.zip › S20_Table.pdf]

S20 Table 13. Purpose and mortality (no covariates).

	**Model 0 (excludes all covariates)**
	HR (95% CI)
Period: 2006-2010 (years 1-4, *n*=5,953 individuals and 600 deaths)	
1.00-2.99	**6.90 (3.89-12.22)**
3.00-3.99	**3.64 (2.14-6.19)**
4.00-4.99	**2.35 (1.39-3.99)**
5.00-5.99	1.14 (0.65-1.98)
6.00 (reference)	1
Period: 2010-2014 (years 5-8, *n*=5,221 individuals and 702 deaths)	
1.00-2.99	**3.32 (1.86-5.94)**
3.00-3.99	**3.65 (2.26-5.87)**
4.00-4.99	**1.91 (1.19-3.09)**
5.00-5.99	**1.72 (1.06-2.78)**
6.00 (reference)	1
Period: 2014-2018 (years 9-12, *n*=4,217 individuals and 756 deaths)	
1.00-2.99	1.51 (0.87-2.63)
3.00-3.99	**2.15 (1.47-3.13)**
4.00-4.99	1.33 (0.92-1.94)
5.00-5.99	1.01 (0.69-1.48)
6.00 (reference)	1

This table reports hazard ratios from Cox models for years 1-4 (2006-2010), 5-8 (2010-2014), and 9-12 (2014-2018). The hazard ratios are for unadjusted purpose categories (i.e., the model excludes all other covariates). Boldface indicates statistical significance ($p<0.05$).
