# Supplementary material for: The importance of baseline health in linking life purpose to longevity
Source: PLoS One. 2026 May 21;21(5):e0349401. doi: 10.1371/journal.pone.0349401 (PMC13193554; doi:10.1371/journal.pone.0349401)
Supplement: S1 File — S2 Fig 1. Data cleaning flowchart. S3 Table 1. Censored and death 2006–2010. S4 Table 2. Censored and death 2010–2014. S5 Table 3. Censored and death 2014–2018. S6 Text 1. Baseline health variable construction. S7 Table 4. Variable definitions and sources. S8 Table 5. Descriptive characteristics of 2006 HRS participants. S9 Table 6. Hazard ratios for individual chronic diseases from Model 3. S10 Table 7. Factor loadings for broad limitations measure. S11 Table 8. Model 2 sensitivity of baseline health to inclusion of purpose. S12 Table 9. Model 3 sensitivity of baseline health to inclusion of purpose. S13 Table 10. Model 4 sensitivity of baseline health to inclusion of purpose. S14 Table 11. Constant proportionality tests. S15 Fig 2. Schoenfeld residual plots for life purpose score. S16 Text 2. Absolute risks. S17 Fig 3. Absolute risks for life purpose. S18 Text 3. Continuous life purpose. S19 Table 12. Continuous life purpose and mortality. S20 Table 13. Purpose and mortality (no covariates). S21 Text 4. The role of multicollinearity. S22 Table 14. Models 6–9 (adding health metrics one at a time). S23 Table 15. Standard errors for purpose (Models 0–9). S24 Table 16. Variance inflation factors (Models 0–9). S25 Table 17. Variance inflation factors for individual purpose categories. S26 Table 18. Variance inflation factors for purpose. S27 Text 5. Updating purpose and/or health. S28 Table 19. Model 3 updated purpose or updated baseline health. S29 Table 20. Models 1 and 3 with updated purpose and baseline health. S30 Table 21. Model 2 (includes participants without additional health metrics). S31 Table 22. Model 5—Adding psychological status variables to Model 4. S32 Text 6. Mortality in years 1–2 and 3–4. S33 Table 23. Life purpose and mortality (years 1–2 versus 3–4). S34 Text 7. Analysis by chronic condition and age. S35 Table 24. Models 1 and 3 for those with and without chronic condition. S36 Table 25. Models 1 and 3 (continuous purpose) for those with and witho [file pone.0349401.s001.zip › S21_Text.pdf]

S21 Text 4. The role of multicollinearity.

One potential concern is that the attenuation in the role of purpose when adding multiple measures of baseline health (e.g., our Models 3 or 4) arises because multicollinearity inflates standard errors causing the coefficients associated with purpose to be estimated with greater error. Recognize, however, that multicollinearity does not result in biased coefficient estimates (i.e., multicollinearity is as likely to inflate as deflate point estimates). Multicollinearity, however, does cause less precision in estimates and, perhaps, in this case, resulted in smaller point estimates for the less precisely measured purpose coefficients. There are, however, at least three concerns with that interpretation. First, such an interpretation is inconsistent with the literature's conclusion that the relation between purpose and longevity remains strong when controlling for baseline health. Second, the early mortality exclusion also generates dramatic attenuation in the hazard ratio associated with low purpose regardless of the health metrics added.

Third, and most important, there is no evidence that multicollinearity drives the attenuation. Specifically, we conduct a series of five tests to directly investigate how simultaneously entering the additional health metrics into the model impacts the purpose estimates due to additional multicollinearity.

1. We begin by examining the analysis with four new models (Models 6-9) that replaces the Model 2 health variable (functional score) with each of the new health metrics individually: broad limitations (Model 6), lung function (Model 7), grip strength (Model 8) and self-rated health (Model 9). Results of these tests, along with results for Models 1 (with no health metrics) and Model 2 (the model from Alimujiang et al. [3]) for ease of comparison, are reported in S22 Table 14. Note in all cases, we follow Model 2 and include an indicator for any chronic disease (rather than indicators for each chronic disease as in our Models 3 and 4). The results are easily summarized. In the initial 4-year period, adding any health metric attenuates the relation between purpose and longevity (i.e., compare Model 1 to Models 2, 6, 7, 8, and 9). Second, once imposing an early mortality exclusion, there is little evidence of a systematic relation between the purpose categories and longevity regardless of whether the model includes no health metrics (Model 1) or which individual health metric is introduced (Models 2, 6-9). Third, when included as the only metric, every health measure is meaningfully related to longevity in years 1-4 and 5-8. For years 9-12, only functional score fails to meaningfully predict longevity. Fourth, although each health metric attenuates the effect of life purpose (i.e., compare each to Model 1), the degree of attenuation is relatively constant consistent with the hypotheses that the measures capture different dimensions of health or the measures are redundant (implying substantial multicollinearity at least between the health measures).

2. Given the issue with multicollinearity is inflated standard errors (rather than biased coefficients), we first examine the role of multicollinearity by examining how adding additional explanatory variables affects the standard errors of the purpose estimates. If multicollinearity is responsible for the attenuation, then the standard errors associated with purpose should increase substantially when multiple measures of baseline health are simultaneously introduced to the model. S23 Table 15 reports the standard errors associated with the four life purpose coefficients from ten models: Model 0 which includes only the life purpose categories (and no additional covariates; see S20 Table 13), Models 1-4 are the corresponding models reported in Table 1, Model 5 adds psychological dimensions to Model 4 (see S31 Table 22), and Models 6-9 that replace function score in Model 2 with each of the additional health metrics individually as detailed in the previous paragraph. The results reveal no evidence that multicollinearity decreases the precision of the estimated coefficients associated with life purpose when moving across the ten models. For example, the average standard error of the four purpose categories increases less than 1.4% when moving from Model 1 (which includes no health measures) to Model 4 (that includes all the health measures simultaneously: indicators for individual chronic disorders, functional score, broad limitations, lung function, grip strength, and self-rated health).

3. Multicollinearity is typically investigated via the estimation of variance inflation factors (VIFs). Recall, a VIF of 1 implies a variable is orthogonal to all other predictors in the model. As a rule of thumb, a VIF between 1-5 is generally viewed as acceptable (i.e., relatively minor multicollinearity), values larger than 5 are viewed as indicating substantial multicollinearity, and values greater than 10 are viewed as problematic. S24 Table 16 reports VIFs for the ten models (see previous paragraph for details) and reveals three insights. First, and most important, variation in VIFs across the models is negligible. That is, the degree of multicollinearity is similar across *all* of the models. For example, the average VIF across the four life purpose coefficients increases by less than 0.05 (from 3.48 to 3.53) when adding all our health metrics (Model 4: individual chronic disorders, the broad limitations metric, lung function, grip strength, and self-rated health) to the Alimujiang et al. (2019) model (Model 2). Second, all VIFs are less than 5, suggesting acceptable levels of multicollinearity. Third, although all the VIFs are less than 5, the VIFs for life purpose between 4.00-4.99 and 5.00-5.99 are between 4 and 5 in all ten models, suggesting we are getting close to the rule of thumb limit. The (relatively) high VIFs for the purpose categories arise, however, because if one knows three of the remaining four categories, one can infer that p9urpose is either in the excluded group (purpose=6) or the remaining category. For example, the VIFs for Model 0, that *only* includes the purpose categories, are nearly identical to the corresponding VIFs for Model 4 that incorporates all the baseline health metrics.

4. As a further test, we also estimate VIFs, for each purpose category, that include all the explanatory variables in Model 4 (the model that includes all the baseline health metrics simultaneously) *except the other purpose categories*. That is, we remove the multicollinearity between the purpose categories, and examine, for each purpose category individually, variance inflation due to all other variables in the model. These results, reported in S25 Table 17, reveal that once excluding the other purpose categories, the VIFs for the most expansive model are uniformly less than 1.1.

5. As detailed in S18 and S19, we also estimate models using life purpose scores rather than the life purpose categories used by Alimujiang et al. [3]. S26 Table 18 reports VIFs associated with the life purpose variable for Models 1-9. The largest VIF (Model 5 which includes the psychological covariates) is less than 1.6. The largest VIF for the models that are the focus of our study (Models 1-4) is 1.2.
