# Supplementary material for: The importance of baseline health in linking life purpose to longevity
Source: PLoS One. 2026 May 21;21(5):e0349401. doi: 10.1371/journal.pone.0349401 (PMC13193554; doi:10.1371/journal.pone.0349401)
Supplement: S1 File — S2 Fig 1. Data cleaning flowchart. S3 Table 1. Censored and death 2006–2010. S4 Table 2. Censored and death 2010–2014. S5 Table 3. Censored and death 2014–2018. S6 Text 1. Baseline health variable construction. S7 Table 4. Variable definitions and sources. S8 Table 5. Descriptive characteristics of 2006 HRS participants. S9 Table 6. Hazard ratios for individual chronic diseases from Model 3. S10 Table 7. Factor loadings for broad limitations measure. S11 Table 8. Model 2 sensitivity of baseline health to inclusion of purpose. S12 Table 9. Model 3 sensitivity of baseline health to inclusion of purpose. S13 Table 10. Model 4 sensitivity of baseline health to inclusion of purpose. S14 Table 11. Constant proportionality tests. S15 Fig 2. Schoenfeld residual plots for life purpose score. S16 Text 2. Absolute risks. S17 Fig 3. Absolute risks for life purpose. S18 Text 3. Continuous life purpose. S19 Table 12. Continuous life purpose and mortality. S20 Table 13. Purpose and mortality (no covariates). S21 Text 4. The role of multicollinearity. S22 Table 14. Models 6–9 (adding health metrics one at a time). S23 Table 15. Standard errors for purpose (Models 0–9). S24 Table 16. Variance inflation factors (Models 0–9). S25 Table 17. Variance inflation factors for individual purpose categories. S26 Table 18. Variance inflation factors for purpose. S27 Text 5. Updating purpose and/or health. S28 Table 19. Model 3 updated purpose or updated baseline health. S29 Table 20. Models 1 and 3 with updated purpose and baseline health. S30 Table 21. Model 2 (includes participants without additional health metrics). S31 Table 22. Model 5—Adding psychological status variables to Model 4. S32 Text 6. Mortality in years 1–2 and 3–4. S33 Table 23. Life purpose and mortality (years 1–2 versus 3–4). S34 Text 7. Analysis by chronic condition and age. S35 Table 24. Models 1 and 3 for those with and without chronic condition. S36 Table 25. Models 1 and 3 (continuous purpose) for those with and witho [file pone.0349401.s001.zip › S27_Text.pdf]

S27 Text 5. Updating purpose and/or health.

One potential concern is purpose can change over time and therefore the early mortality exclusion attenuates the purpose-longevity relation because purpose at baseline is not identical to purpose 4 (or 8) years later. Of course, by symmetry, the same argument holds for health. Nonetheless, one recent study [4] takes such an approach and while updating the life purpose metric uses lagged measures of baseline health. Such an approach mechanically increases measurement error in baseline health resulting in greater residual confounding. For example, if a Stage IV brain cancer diagnosis (in the period between when lag health and updated purpose are measured) increases mortality risk and decreases the extent to which a person enjoys '…making plans for the future and working to make them a reality,' then updated purpose will, to some extent, proxy for the excluded variable updated health. In short, updating only purpose or baseline health without updating the other leads to a bias favoring the updated variable—as the updated variable will better capture baseline health at the start of the evaluation period.

In sum, updating only purpose or baseline health without updating the other leads to a bias favoring the updated variable—as the updated variable will better capture baseline health at the start of the evaluation period. That is, unless one is willing to assume that changes in health have no impact on changes in purpose, updating purpose without updating health increases residual confounding. Consistent with our hypothesis, when updating one variable but not the other, the updated variable performs "better." Specifically, S28 Table 19 reports values using updated health but *lag* purpose (columns 1 and 2) or updated purpose but *lag* health (columns 3 and 4). Health performs better (i.e., hazard ratios for health have a larger impact in columns 1-2 than 3-4) when health is updated but purpose is lagged. Correspondingly, the hazard ratios associated with purpose increase when using updated purpose but lagged health (i.e., hazard ratios for purpose have a larger impact in columns 3-4 than columns 1-2).

Given updating purpose while using lag health mechanically increases the residual confounding, the alternative approach is to use updated values for both purpose and health—which, of course, just repeats the tests in the top portion of Table 1 (over different sample periods) examining whether purpose predicts longevity over the initial 4-year period when better controlling for baseline health. S29 Table 20 reports hazard ratios from Cox models for the three 4-year periods with updated covariates: (1) 2006-2010, all variables measured in 2006 (i.e., identical the results in the top portion of Table 1), (2) 2010-2014, all variables measured in 2010, and (3) 2014-2018, all variables measured in 2014. Because the bottom two portions of the table simply repeat the tests in the top of the table with different samples, it is not surprising the results are nearly identical across the top, middle, and bottom portion of the table. In short, we reach the same conclusion—adding additional dimensions of baseline health greatly attenuates the relation between purpose and longevity, even absent an early mortality exclusion.
